# Sulfur-cycling chemolithoautotrophic microbial community dominates a cold, anoxic, hypersaline Arctic spring

**DOI:** 10.1186/s40168-023-01628-5

**Published:** 2023-09-11

**Authors:** Elisse Magnuson, Ianina Altshuler, Nastasia J. Freyria, Richard J. Leveille, Lyle G. Whyte

**Affiliations:** 1 Natural Resource Sciences, McGill University, Ste-Anne-de-Bellevue, QC Canada; 2 MACE Laboratory, ALPOLE, School of Architecture, Civil and Environmental Engineering (ENAC), Ecole Polytechnique Fédérale de Lausanne, Lausanne, Switzerland; 3 Department of Earth and Planetary Sciences, McGill University, Montreal, QC Canada; 4 Geosciences Department, John Abbott College, Ste-Anne-de-Bellevue, QC Canada

**Keywords:** Metagenomics, Metatranscriptomics, Metagenome-assembled genomes, Saline spring, Sulfidic spring, Cryosphere

## Abstract

**Background:**

Gypsum Hill Spring, located in Nunavut in the Canadian High Arctic, is a rare example of a cold saline spring arising through thick permafrost. It perennially discharges cold (~ 7 °C), hypersaline (7–8% salinity), anoxic (~ 0.04 ppm O_2_), and highly reducing (~ − 430 mV) brines rich in sulfate (2.2 g.L^−1^) and sulfide (9.5 ppm), making Gypsum Hill an analog to putative sulfate-rich briny habitats on extraterrestrial bodies such as Mars.

**Results:**

Genome-resolved metagenomics and metatranscriptomics were utilized to describe an active microbial community containing novel metagenome-assembled genomes and dominated by sulfur-cycling *Desulfobacterota* and *Gammaproteobacteria*. Sulfate reduction was dominated by hydrogen-oxidizing chemolithoautotrophic *Desulfovibrionaceae* sp. and was identified in phyla not typically associated with sulfate reduction in novel lineages of *Spirochaetota* and *Bacteroidota*. Highly abundant and active sulfur-reducing *Desulfuromusa* sp. highly transcribed non-coding RNAs associated with transcriptional regulation, showing potential evidence of putative metabolic flexibility in response to substrate availability. Despite low oxygen availability, sulfide oxidation was primarily attributed to aerobic chemolithoautotrophic *Halothiobacillaceae*. Low abundance and transcription of photoautotrophs indicated sulfur-based chemolithoautotrophy drives primary productivity even during periods of constant illumination.

**Conclusions:**

We identified a rare surficial chemolithoautotrophic, sulfur-cycling microbial community active in a unique anoxic, cold, hypersaline Arctic spring. We detected Mars-relevant metabolisms including hydrogenotrophic sulfate reduction, sulfur reduction, and sulfide oxidation, which indicate the potential for microbial life in analogous S-rich brines on past and present Mars.

Video Abstract

**Supplementary Information:**

The online version contains supplementary material available at 10.1186/s40168-023-01628-5.

## Background

The cold saline springs on Axel Heiberg Island (AHI), located in the High Arctic in Nunavut, Canada, are rare examples of non-volcanic perennial springs discharging in an area of continuous permafrost. The Gypsum Hill (GH) springs (79° 24′ 30″ N, 90° 43′ 05″ W) are a collection of approximately 40 springs and seeps located on the bank of Expedition River. These springs flow to the surface through 500–600 m of permafrost in an area of gypsum-anhydrite diapiric uplift, and are suggested to originate in a subsurface salt layer recharged by an ice-covered alpine lake or sub-glacial meltwater [1]. They are characteristically cold (~ − 1.3–7.2 °C) and hypersaline (7–8% salinity) and maintain stable conditions year-round despite average air temperatures of − 15 °C, reaching down to − 40 °C in the winter [2]. Sediments and waters in the largest spring outlet, referred to as GH-4, are anoxic (~ 0.04 ppm O_2_), highly reducing (~ − 430 mV oxidation–reduction potential), saturated in dissolved gases (99% N_2_), and rich in sulfate (1.9 g.kg^−1^ and 2.2 g.L^−1^ respectively) and sulfide (9.5 ppm) [3, 4].

The AHI saline springs have been described extensively as analogs to sulfurous, cold, saline environments on extraterrestrial bodies such as Mars [1, 5–7]. As warm, wet ancient Mars became colder and drier during the Hesperian (~ 3.7 Gya), the last surface waters were likely cold brines persisting up to 2 Gya [8]; sulfate salt enrichments in the former lake basin Gale Crater indicate the presence of widespread sulfate-rich brines during this time [9]. The modern Martian surface and subsurface are also rich in Mg- and Ca-sulfates [10, 11], commonly occurring up to 10 wt% [12] and reaching 50 wt% in Gale Crater enrichments [9]. While the atmospheric pressure and temperature on present-day Mars prevents the formation of surficial standing liquid water, there are potential sources of liquid water in the Martian subsurface. Recent evidence suggests the presence of hypersaline lakes below Mars’ southern ice cap [13]. Another possible source of liquid water is the recurring slope lineae; recent modeling suggests they may form from near-surface chloride and sulfate salt brines [14]. Geomorphological features resembling gullies formed in the recent geologic past [15] are suggested to have formed from subsurface eutectic brines [16]. Thus, the GH springs provide a useful analog for examining microbial diversity in a sulfate-rich cryoenvironment such as those that may exist or have existed on Mars.

Cycling of inorganic sulfur compounds sustains microbial life in sulfur-rich terrestrial environments including hydrothermal vents [17, 18], marine sediments [19], and sulfidic springs [20, 21]. In the cryosphere, chemolithoautotrophic S-cycling microbes drive primary productivity in a High Arctic sulfur-rich supraglacial spring at Borup Fjord Pass, Ellesmere Island [22, 23], sulfide-rich thermal springs in Svalbard, Norway [22, 24], and sulfate-rich subglacial brines at Blood Falls, Antarctica [25], and have been detected in cold hypersaline environments such as cryopegs [26] and Antarctic lake brines [27]. Microbial communities at other hypersaline AHI springs contain abundant sulfur-oxidizing *Gammaproteobacteria* and sulfate-reducing *Desulfobacterota* (formerly *Deltaproteobacteria*) [6, 28]. However, microbial community sequencing in many of these cryospheric S-cycling environments, including GH, has been limited to 16S rRNA gene community profiling or 16S rRNA gene sequencing of isolates. Recent high-throughput shotgun metagenomic and metatranscriptomic sequencing of nearby hypersaline, sub-zero Lost Hammer Spring revealed previously undetected bacterial and archaeal species and identified active sulfide-oxidizing *Gammaproteobacteria* driving primary production [28], demonstrating the power of these sequencing approaches in describing sulfur cycling and microbial diversity in these cryospheric Mars analogs. Detection of active microorganisms through approaches such as metatranscriptomic or metaproteomic sequencing are especially crucial in open-system anoxic, cold, hypersaline environments like GH, where dormant or dead populations may be present and organic matter including DNA may be preserved [5]. Indeed, detected active microbial communities at AHI springs including Lost Hammer differ from the total microbial biomass [6, 28, 29].

Previous studies of the GH sediment community indicate the presence of an active microbial community (10^6^–10^7^ cells.g^−1^ sediment) [3, 4, 30]. Sequencing of 16S rRNA genes identified *Gammaproteobacteria* and *Desulfobacterota* phylotypes associated with sulfur cycling [3, 4, 31, 32], and microcosm activity measurements and isotopic analyses indicate sulfur oxidation and sulfate reduction occur in the GH spring sediments [4, 32, 33]. However, no studies have yet fully described the taxonomic and metabolic diversity present in the sediment or definitively linked detected metabolic activities to active taxa in situ. As such, this study utilized genome-resolved metagenomics and metatranscriptomics to identify the primary active microbial community in the springs and characterize sulfur-cycling metabolisms and taxa in depth in the context of the GH springs as a unique cryospheric analog to sulfate-rich environments on Mars.

## Methods

### Site description and sampling

As described in previous publications [1–4], the GH springs emerge in approximately 40 outlets across a 300-m length of the Expedition River bank, 2.5 km downstream from the White and Thompson glaciers. This region experiences seasonal darkness from ~ October to February. Gypsum (CaSO_4_), halite (NaCl), and elemental sulfur are the most abundant minerals across the GH site [5]. The spring outlet described in this study is referred to here and in previous publications as GH-4. At GH-4, spring water emerges from the subsurface into a primary pool, measuring ~ 1.7 m in diameter with a depth of ~ 45 cm, then flows into a shallow outflow channel (Figure S1). Dissolved gases bubble continuously through the outflow pool sediment and water and are predominantly comprised of N_2_ (~ 99%) with low levels of CO_2_ (0.04%) and CH_4_ (0.5%) [4, 34]. All spring samples and measurements described in this study were taken in the primary outflow pool of GH-4.

Physical and chemical parameters in GH-4 have remained highly stable since 2007 (Table S1), allowing for comparison of samples collected in different years. Sediment samples (top ~ 10 cm) for metagenomic and metatranscriptomic sequencing were collected in July 2019, and sediment samples for 16S rRNA gene community profiling were collected in August 2021. All sediment samples were collected with a sterilized scoop and mixed immediately with Zymo Research DNA/RNA Shield (Irvine, CA, USA) in a sterile Falcon tube filled to maximum to avoid aerobic headspace. Samples were frozen at − 20 °C after sampling, then kept at < 7 °C during transport to Montréal where they were again stored at − 20 °C prior to processing. Thermochron Temperature Loggers (Baulkham Hills, NSW, Australia) were used to track temperature conditions during transport.

Water temperature, pH, oxidation–reduction potential, salinity, and total dissolved solids were measured in situ with a YSI Professional Plus Multiparameter Instrument (Yellow Springs, OH, USA). Dissolved oxygen was measured in situ with a PyroScience Piccolo2 oxygen meter (Aachen, Germany). Sulfide, sulfate, nitrite, nitrate, phosphate, ammonia, and iron were measured in situ with CHEMetrics Inc. (Midland, VA, USA) test kits. Water for total carbon and total nitrogen measurements was filtered immediately after sampling with a 0.22-µm Whatman Uniflo PES filter (Madstone, UK). Filtered water was stored at − 20 °C and thawed at 4 °C prior to analysis on a Shimadzu TOC-VCPH Total Organic Carbon Analyzer with TNM-L Total Nitrogen Measuring Unit (Kyoto, Japan).

### DNA extraction, metagenome sequencing, and metagenome analyses

DNA was extracted from 3 × 10 g portions of sediment with the Qiagen DNeasy PowerMax Soil Kit (Hilden, Germany). Extracted DNA was concentrated with a Thermo Fisher Scientific SpeedVac Vacuum Concentrator (Waltham, MA, USA) and New England Biolabs Monarch PCR & DNA Cleanup Kit (5 µg) (Ipswitch, MA, USA). Sequencing libraries were prepared with the Illumina Nextera XT DNA Library Prep Kit and sequenced at The Center for Applied Genomics at the Hospital for Sick Children (Toronto, ON, Canada) on a NovaSeq 6000 (Illumina) with an S Prime 100-cycle flow cell (2 × 100 base pairs).

Metagenome sequencing produced ~ 109 million reads across the three replicates (sequencing and assembly statistics in Table S2). Read quality was checked before and after quality control with FastQC (v.0.11.9). Adapters and reads were trimmed with BBDuk (BBMap v.38.96) with parameters trim = r k = 23 mink = 11 hdist = 1 tpe tbo ftm = 5. PhiX contamination was removed with BBDuk using the BBMap PhiX174 reference genome (parameters k = 31 hdist = 1), and human genome contamination was removed with the BBMap Removehuman.sh script with default parameters using the BBMap GH19 masked human reference genome. After contaminant filtering, all reads < 30 bp were removed with BBDuk (minlength = 30). Remaining reads were classified with Kaiju (v.1.7.4) [35] with the nr_euk reference database and with phyloFlash (v.3.4) [36].

The three replicate metagenomes were co-assembled with Megahit (v.1.2.9) using three pre-set settings (default, meta-sensitive, and meta-large) [37]. The “meta-sensitive” assembly was selected for further analysis based on assembly quality as assessed with MetaQUAST (v.5.0.2) [38]. Co-assembly statistics are compiled in Table S3. The co-assembly was annotated with the Joint Genome Institute IMG/M Annotation Pipeline (v.5.1.5) using KEGG, COG, and TIGRFAM databases [39, 40]. Reads were mapped to the assemblies with Bowtie2 (v.2.4.4) (–very-sensitive) [41] and assemblies were binned with MetaBAT (v.2.14) [42] and MaxBin (v.2.2.7) [43] with minimum contig sizes of 1000, 2000, 2500, and 5000 bp. Bin completeness and contamination were assessed with CheckM (v.1.1.9) [44], and bin taxonomy was assigned with GTDB-Tk (v.2.1.1, reference package R207-v2) [45]. A minimum contig size of 2000 bp was selected based on quantity of high-quality bins. Assemblies were additionally binned with CONCOCT (v.1.1.0) (–length_threshold 2000) [46]. MetaBAT, MaxBin, and CONCOCT bins were de-replicated with DAS Tool [47] to create a set of non-redundant bins, and bin contamination was further reduced where possible with RefineM (v.0.0.25) [48].

An additional binning strategy adapted from Chen et al. [49] was also used for comparison to ensure the highest bin quality possible. Briefly, the three replicate metagenomes were assembled individually with Megahit and SPAdes (v.3.15.3) (–meta). The SPAdes metagenomes were selected for further analysis based on assembly quality. Read mapping, binning, dereplication, and bin refinement were performed as above to produce a set of non-redundant bins for each metagenome. Following this, the bins from all three metagenomes were pooled and again de-replicated with dRep (v.3.2.2) [50], resulting in a final set of de-replicated bins. After comparison between the two resultant bin sets, the co-assembly bins were selected for all downstream analyses based on prevalence of high-quality bins.

Additional analyses were as follows: Bin abundance in the metagenome was calculated with CoverM (v.0.4.8). A phylogenomic tree of MAGs was created in anvi’o (v.6.2) [51] with the Bacteria_71 collection of single-copy genes. Amino acid sequences for all genes were concatenated, with a total alignment length of 23,122 bp, and approximately-maximum-likelihood trees were constructed with FastTree (v.2.1) [52] within anvi’o with midpoint rooting. FeGenie (v.1.2) was used to identify iron-related genes [53]. Hydrogenases were classified with hydDB [54]. Reductive and oxidative DsrAB were classified as follows: DsrAB amino acid sequences were aligned against reference sequences from Müller et al. [55] using MUSCLE (v.3.8.1551) with default settings [56]. Maximum likelihood phylogenetic trees were constructed in CLC Genomics Workbench (v.12.0.3; QIAGEN, Aarhus, Denmark) using the WAG protein substitution model and 1000 bootstraps (Figures S2, S3, S4, S5). DsrAB were classified as reductive or oxidative based on phylogenetic clustering and the presence of accessory proteins as in Anantharaman et al. [57].

### mRNA extraction, metatranscriptome sequencing, and analyses

RNA was extracted from 3 ×  ~ 6 g sediment samples with the Zymo Research ZymoBIOMICS DNA/RNA Miniprep kit. Each replicate was split into six sub-samples for the extraction. Extracted RNA was treated with the Invitrogen Turbo DNA-free kit (Carlsbad, CA, USA) to remove contaminating DNA. The six sub-samples in each replicate were then pooled together and concentrated with the New England Biolabs Monarch RNA Cleanup Kit. Extractions were checked for DNA contamination with a Thermo Fisher Scientific Qubit dsDNA HS Assay Kit and by PCR amplification of 16S rRNA genes using DNA primers 27F/1492R [58, 59]; only samples with no amplified product (as detected by agarose gel electrophoresis) were used. Ribosomal RNA was depleted with the New England BioLabs NEBNext rRNA Depletion Kit (Bacteria) and a cDNA sequencing library was prepared with the New England BioLabs Ultra II RNA Library Prep Kit. For one extraction replicate (GH3), two sequencing libraries were prepared from the extraction, with one library undergoing fragmentation (R9) and one not fragmented (R6), in order to optimize library quality based on its RNA Integrity Number. The generated libraries were sequenced at The Center for Applied Genomics at the Hospital for Sick Children on a NovaSeq 6000 (Illumina) with an S Prime 100-cycle flow cell (2 × 100 base reads).

Metatranscriptome library statistics are compiled in Table S2. Adapter trimming and PhiX and human contamination removal was done with BBDuk as for the metagenomic reads. Additional quality trimming was done with BBDuk (qtrim = r trimq = 15 maq = 15 minlen = 50). Remaining rRNA reads were removed with SortMeRNA (v.4.3.4) [60]. The trimmed reads were classified with Kaiju and mapped to the co-assembly contigs with bowtie2 (–very-sensitive). Reads aligned to metagenome features were counted with htseq-count (-s no-i ID –nonunique = all -r pos -a 0), and transcripts per million reads (tpm) was calculated to normalize transcript abundance for each gene. Counts from the two technical replicates (R6 and R9) were merged prior to tpm calculation. Normalized transcript abundance values represent averages of the three biological replicates unless otherwise noted.

### 16S rRNA gene community profiling and analyses

DNA was extracted from 3 × 1 g sediment samples with the Zymo Research ZymoBIOMICS DNA/RNA Miniprep kit. Two negative controls (nuclease-free water) and two replicates of the ZymoBIOMICS Microbial Community Standard were also extracted. All extractions were concentrated with the New England Biolabs Monarch PCR & DNA Cleanup kit. Concentrated DNA was amplified by PCR with 16S rRNA gene V4 primers 515F-Y and 926R [61] with Illumina overhang adapters [62]. Each 25 µL reaction contained 10 µL Qiagen HotStarTaq Plus Master Mix, 0.5 µL each of 10 µM forward and reverse primers, 1 µL 10 µg.µL^−1^ bovine serum albumin, 5 µL extracted DNA, and 3 µL nuclease-free water. PCR cycling proceeded as follows: 5 min at 95 °C; 35 cycles of 45 s at 94 °C, 45 s at 50 °C, and 1 min at 72 °C; and 10 min at 72 °C. Amplicon sequencing libraries were prepared according to the Illumina 16S Metagenomic Sequencing Library Preparation protocol [62]. Briefly, PCR products were cleaned using Cytiva Sera-Mag Select (Marlborough, MA, USA) magnetic beads at a ratio of 0.6 × beads:sample volume. Nextera XT Index Kit v2 Set B dual indices were attached by PCR in 50 µL reactions containing 25 µL Invitrogen Platinum Hot Start PCR 2 × Master Mix (Waltham, MA, USA), 5 µL PCR product, 5 µL each index primers 1 and 2, and 10 µL PCR-grade water. PCR cycling proceeded as follows: 3 min at 95 °C; 8 cycles of 30 s at 95 °C, 30 s at 55 °C, and 30 s at 72 °C; and 5 min at 72 °C. The libraries were again cleaned with Cytiva Sera-Mag Select beads at a ratio of 0.8 × beads:sample volume, then pooled at equimolar concentrations and sequenced on an Illumina MiSeq with MiSeq Reagent Kit v3 (600-cycle) (2 × 300 base reads).

The 16S rRNA gene amplicon sequencing statistics are compiled in Table S2. An amplicon sequence variant (ASV) count table was generated with the DADA2 pipeline (v.1.24.0) [63]. Taxonomy was assigned with the SILVA database (v.138.1) [64]. The decontam R package (v. 1.16.0) was used to identify and remove contaminant ASVs (threshold = 0.5) in addition to manual removal of ASVs present only in the negative controls and the ZymoBIOMICS Microbial Community Standard. In total, 2885 of 2901 ASVs remained after quality control.

The 16S rRNA gene amplicon sequences from GH were compared to 24 additional 16S rRNA gene amplicon sequencing libraries from similar environments (metadata in Table S4). ASV count tables were generated for each library in DADA2 using the SILVA database for taxonomy assignation as above. For single-end libraries (Ion Torrent and 454 metagenomes), the DADA2 pipeline was run with only forward read commands and without read merging. Ion Torrent and 454 metagenomes were also processed with additional parameters as recommended by the DADA2 pipeline (dada(…, HOMOPOLYMER_GAP_PENALTY = -1, BAND_SIZE = 32)). Two data sets did not have sufficient reads to learn error rates (JL94DB and JL95B); for these samples, taxonomy was assigned directly to the reads using the DADA2 assignTaxonomy function without ASV inference. To allow comparison between the data sets, ASV/read counts were summed by taxonomic assignment (Table S5). Dissimilarity and clustering analyses were calculated using the “vegan” Community Ecology package (v.2.6–4) in R. Environmental metadata was standardized with the decostand() function using the “standardize” method. Frequencies were calculated for each taxon by dividing each count by total count per taxon, following by standardization with decostand() using the “hellinger” method. A non-metric multidimensional scaling (NMDS) ordination was generated using the metaMDS() function with Bray–Curtis dissimilarity matrix (dist = ”bray”). A canonical correspondence analysis (CCA) was done using the cca() function with a Bray–Curtis dissimilarity matrix and 999 permutations. CCA was built by automatic forward selection, with adjusted R^2^ to select significant variables. Spearman’s rank correlation was performed using the cor(), rcorr() functions and visualized using corplot() function of the stats, Hmisc and corrgram packages respectively.

## Results and discussion

### Metagenomic sequencing and MAGs characterized taxonomic diversity

**Fig. 1:**
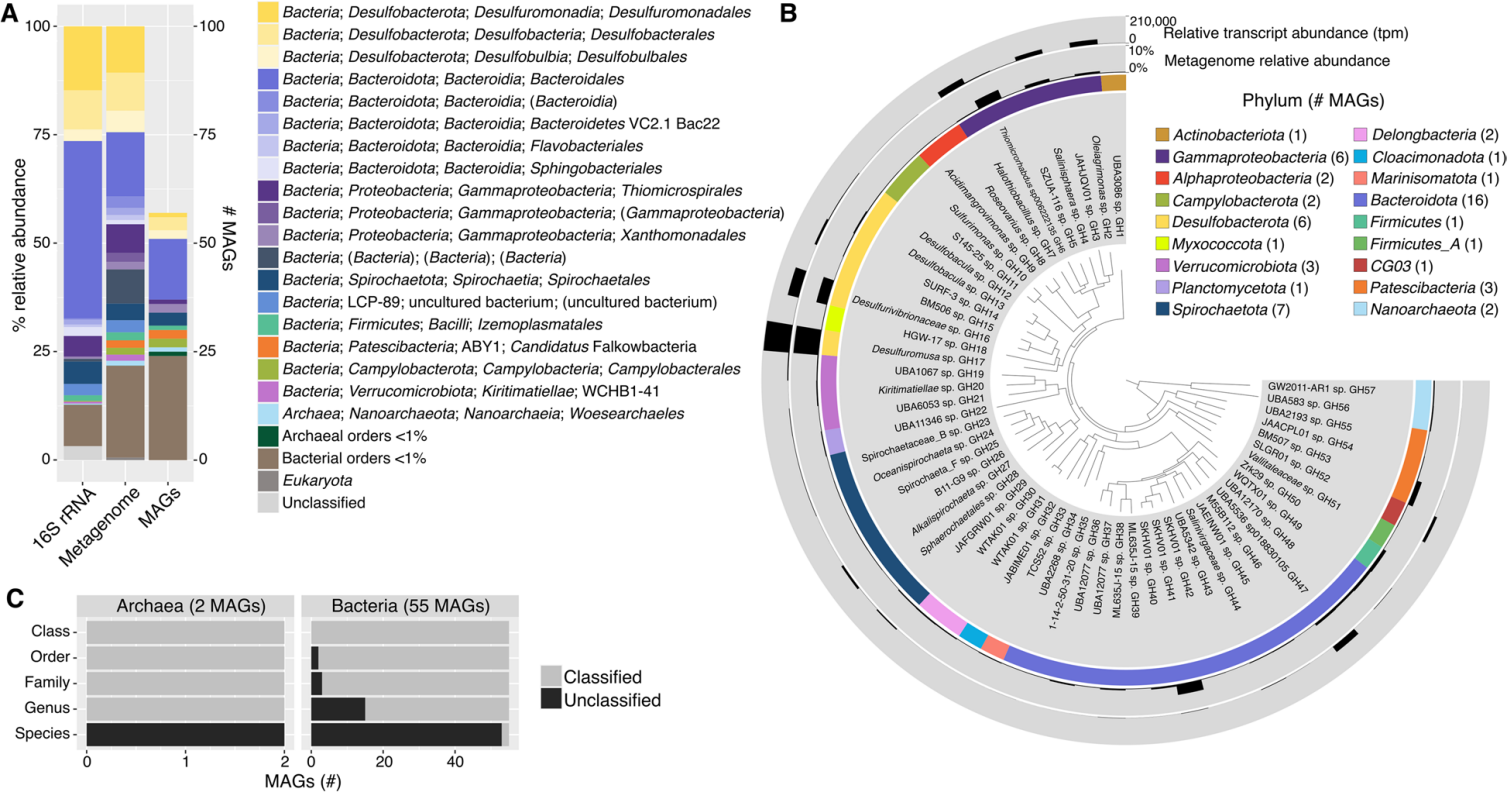
**A** Relative abundance of orders in 16S rRNA gene amplicon sequencing and shotgun metagenomes and number of MAGs in each order. Shotgun metagenome relative abundance was determined by read mapping to SSU rRNA genes with phyloFlash. **B** Phylogenomic tree of the 57 high- and medium-quality MAGs with phylum, relative abundance in the metagenome (percentage of mapped metagenome reads), and relative transcript abundance (transcripts per million reads of genes in each MAG) indicated. The tree was constructed with the anvi’o Bacteria_71 set of single-copy genes for both bacterial and archaeal MAGs and midpoint-rooted. **C** Level of taxonomic novelty of the MAGs. The number of classified and unclassified genomes at each taxonomic level was determined according to its rank assignment and taxonomic placement by GTDB-tk

In order to describe the taxonomic diversity present in the GH sediment, we sequenced 16S rRNA gene amplicons and shotgun metagenomes and constructed metagenome-assembled genomes (MAGs) (Fig. 1). Metagenomic sequencing recovered primarily bacterial reads, with archaeal and eukaryotic reads constituting < 1% (Fig. 1a, Figure S6). The most abundant phyla in the shotgun metagenome and 16S rRNA gene amplicon sequences were *Desulfobacterota* (25 and 26%, respectively), *Bacteroidota* (22 and 46%), *Proteobacteria* (17 and 8.5%) (primarily *Gammaproteobacteria*, 15 and 6.6% of total), and *Spirochaetota* (4.7 and 6.8%). Of the 2885 16S rRNA gene amplicon sequence variants (ASVs), 258 were represented in the metagenome at species-level similarity (> 98.6%); those 258 ASVs comprised 62% of ASV relative abundance, indicating that the majority of ASVs not also present in the shotgun metagenome were likely from low abundance species. Detected taxonomic diversity was similar to previous studies of the GH sediment; of 49 bacterial isolates obtained in 2008 [4], 47 were related at the genus level or above (> 95% sequence identity of 16S rRNA gene) to ASVs from this study. Similarly, 43 of 46 bacterial 16S rRNA gene clone library sequences obtained in 2007 were related at the genus level or above, indicating consistency in the spring microbial community from 2007 to 2019–2021 (this study).

MAG assembly produced 26 high-quality (> 90% complete, < 5% contamination) and 31 medium-quality (> 50% complete, < 10% contamination) bins representing 17 phyla, including bacterial (55 MAGs) and archaeal (2 MAGs) taxa (Fig. 1b; Table S7). Distribution of MAGs across phyla reflected metagenome abundances (Fig. 1a), with the most highly represented phyla being *Bacteroidota* (16 MAGs), *Proteobacteria* (8 MAGs), *Spirochaetota* (7 MAGs), and *Desulfobacterota* (6 MAGs). MAGs were recovered for nearly all of the most abundant orders (> 1.5%) in the metagenome. Taxa above 1.5% relative abundance without representative MAGs were Bacteroidetes VC2.1 Bac22 (ca. Sulfidibacteriales; 1.6%) and LCP-89 (2.8%). Both taxa represent uncultured clades found in oxygen-limited, sulfide-rich environments including marine hydrothermal vents and oxygen-minimum zones (VC2.1 Bac22) and Zodletone Spring in Oklahoma, USA (LCP-89) [65, 66].

MAG novelty was estimated using the taxonomic rank of MAGs classified by comparison to the GTDB database with GTDB-tk [45] (Fig. 1c). The majority of MAGs (97%) were unclassified at the species level, and 26% remained unclassified at higher taxonomic ranks (up to order). Similarly, 40% of ASVs detected by 16S rRNA gene amplicon sequencing were unclassified at genus level or higher (Figure S7), indicating a high level of taxonomic novelty in the GH microbial community.

### Sulfur-cycling taxa and metabolic genes are abundant in the metatranscriptome

**Fig. 2:**
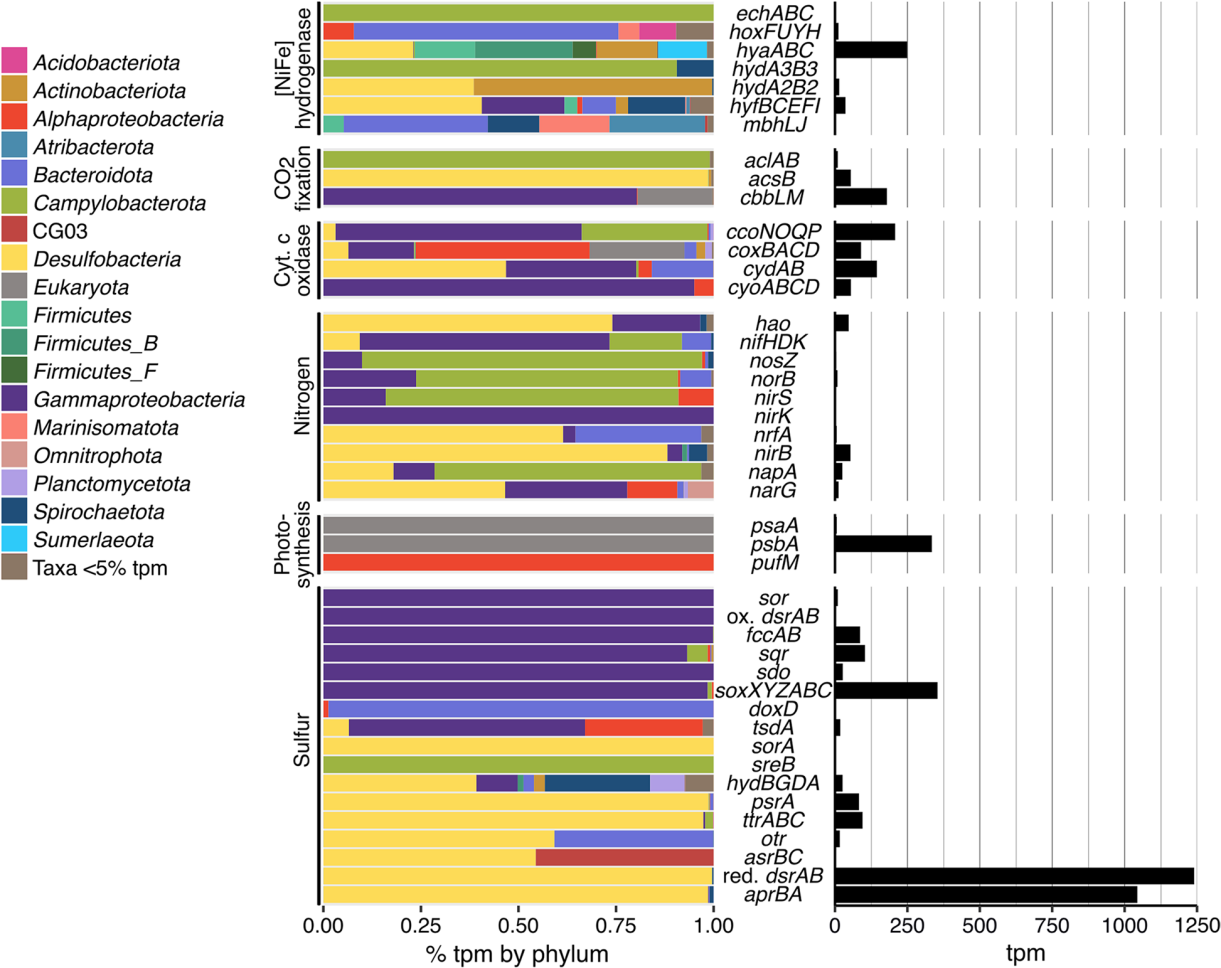
Metabolic gene relative transcript abundance and distribution of transcripts by phylum. Relative transcript abundance by phylum is based on the presence of transcribed genes in MAGs and phylogenetic classification of unbinned genes in JGI. Complete phylogenetic distribution of depicted genes in MAGs is located in Table S8

**Fig. 3:**
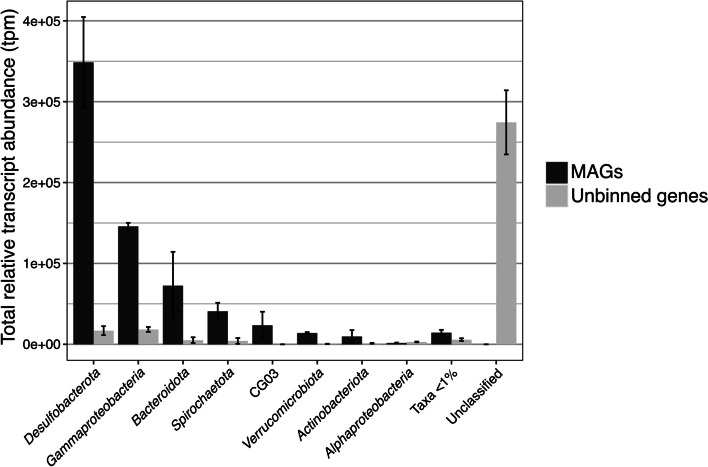
Total relative transcript abundance (tpm) of MAGs and unbinned genes in phyla or classes > 1% relative abundance. Error bars indicate standard deviation between metatranscriptome replicates. Unbinned genes were classified with the JGI Phylo Distribution function

**Fig. 4:**
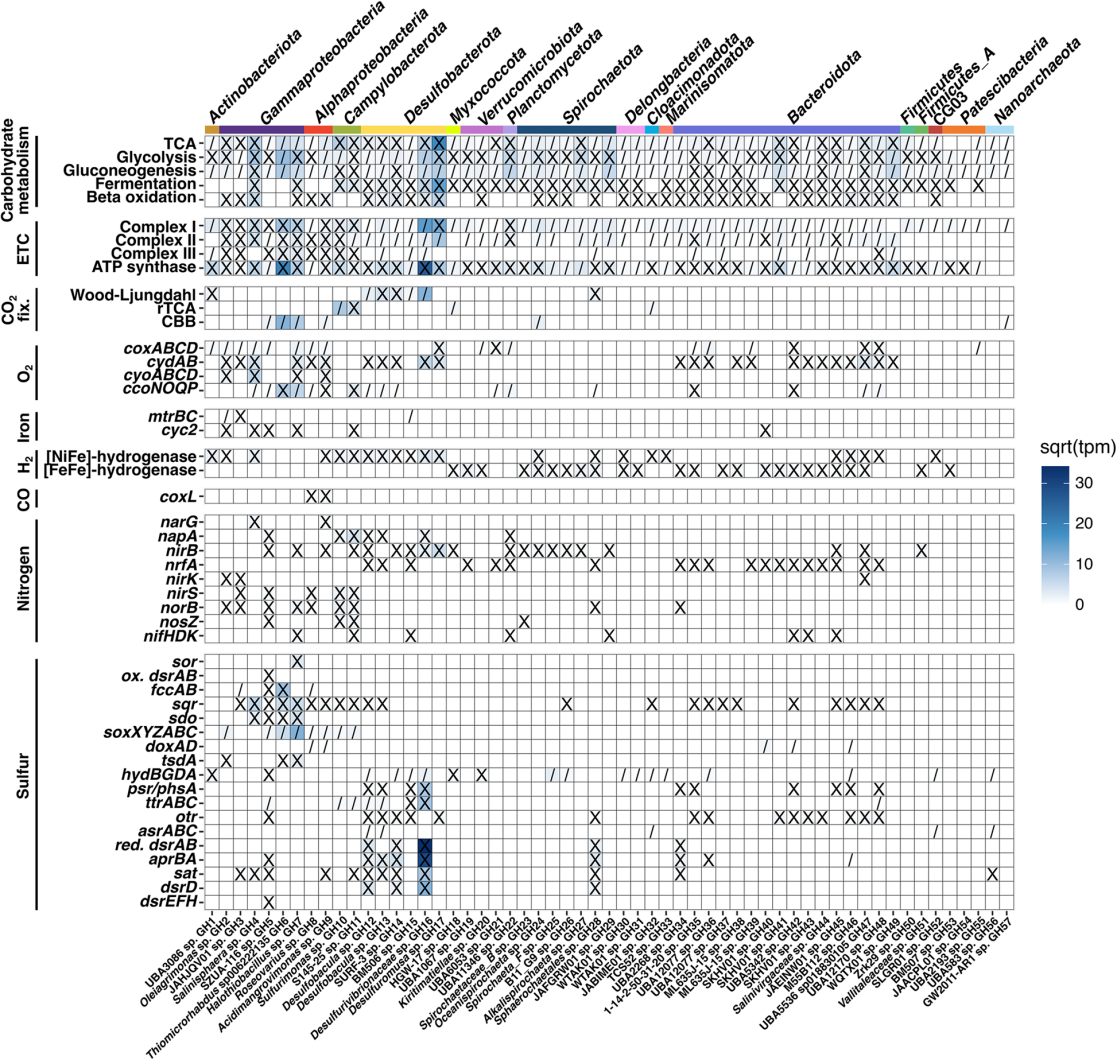
Pathway and gene presence in MAGs. Where applicable, “X” indicates presence of a complete pathway and “/” indicates a partial pathway. Heat map indicates square root of relative transcript abundance (transcripts per million reads) of genes in each MAG. A complete table of gene IDs and criteria for denoting the presence of a complete or partial pathway is located in Table S9; a corresponding table with tpm values is located in Table S10

Metatranscriptomic reads were mapped to the MAGs and metagenomic co-assembly to identify active metabolisms and link detected metabolic activity to microbial taxa. Relative transcript abundance of MAGs (Fig. 1b) and metabolic genes of interest (Fig. 2) indicated that sulfur-cycling metabolisms were predominant in this system. Sulfate is abundant in the spring sediment (1.9 g.kg^−1^) [3] and waters (~ 2200 ppm). Previous isotopic analysis indicated that it is derived from the anhydrite (CaSO_4_) evaporite diapirs that the GH springs flow through in the subsurface [33]. Isotopic analysis also indicated sulfate reduction to sulfide was occurring in the spring sediment; this reducing activity had a quantitatively unimportant impact on fractionation of sulfate flowing into the spring, indicating that sulfate is present in non-limiting quantities for microbial metabolism [33]. Sulfide (~ 9.5 ppm) and other reduced sulfur compounds are also present in non-limiting quantities [4], suggesting favorable conditions for a diversity of sulfur-based microbial metabolisms. Sulfur-cycling taxa were among the most abundant orders in the shotgun metagenome, including *Desulfuromonadales* (11%), *Desulfobacterales* (9%), *Desulfobulbales* (5%), and *Thiomicrospirales* (7%) (Fig. 1a). The highest proportion of relative transcript abundance was attributed to *Desulfobacterota* MAGs (35%), followed by *Gammaproteobacteria* MAGs (14.5%) (Fig. 3); all MAGs in these taxa transcribed sulfur-cycling genes (Fig. 4). Additionally, sulfate-reducing genes (*dsrAB*, 1240 tpm; *aprBA*, 1044 tpm) were the most highly transcribed metabolic genes in the metagenome (Fig. 2; Table S11).

## *H*_*2*_*-linked sulfate reduction drives microbial primary production*

In addition to archetypal sulfate reduction marker genes (*dsrAB, aprBA*), genes involved in tetrathionate (*ttrABC*, *otr*), polysulfide and thiosulfate (*psrA/phsA*), and sulfite (*sreB*, *asrBC*; note that no *asrA* subunits were detected) reduction were also present and transcribed. Sulfur species reduction was relatively widespread; 21 MAGs (37% of total MAGs) had detected transcription of sulfur-reduction-related genes, including *Desulfobacterota*, *Bacteroidota*, *Spirochaetota*, CG03, *Campylobacterota*, and *Gammaproteobacteria* (Fig. 4). This count excludes *hydBGDA* sulfhydrogenase genes, whose direct role in sulfur reduction is considered unlikely but which are highly homologous to a sulfur-reduction-linked membrane-bound oxidoreductase [67, 68].

The majority of S reduction gene transcription (86% of all S reduction gene transcription) mapped to the *Desulfurivibrionaceae* sp. GH16 MAG, primarily due to high transcription of the sulfate reduction genes *dsrAB* (93% of total) and *aprBA* (82%). This MAG was relatively abundant in the metagenome (4%) and transcribed genes for complete sulfate reduction to sulfide (*dsrAB*, *aprBA*, *sat*, *dsrD*; 2238.9 tpm total) [57], as well as tetrathionate (*ttrABC*; 75.5 tpm) and thiosulfate/polysulfide (*phsA*/*psrA*; 68.3 tpm) reduction. It also transcribed genes for dissimilatory nitrate reduction to ammonia (*napA*, 2.3 tpm; *nirB,* 5.6 tpm), indicating flexible use of S and N electron acceptors despite the abundant sulfate in the spring. The MAG also co-transcribed a group 1c [NiFe] hydrogenase involved in hydrogenotrophic respiration (221 tpm) [54], indicating that the majority of sulfate reduction in the spring sediment is coupled with H_2_ oxidation. Previous measurements of spring gas composition have not identified measurable concentrations of H_2_ [4, 34]. However, genes involved in fermentative production of hydrogen are present and transcribed (e.g., formate hydrogenlyase, Table S12), which may represent a putative source of H_2_; thus, hydrogen concentrations may be limiting in sulfate reduction activity.

The *Desulfurivibrionaceae* sp. GH16 MAG could only be classified at the family level in comparison to the GTDB database (Table S7). A partial 16S rRNA gene (846 bp) present in the MAG aligned at 91% identity to an uncultured *Desulfobacterota* when compared to the SILVA database, suggesting that this GH sulfate-reducing bacterium (SRB) likely represents a novel genus or higher lineage of *Desulfobacterota*. Related MAGs within the *Desulfurivibrionaceae* family in the GTDB database were assembled from hypersaline soda lakes (GCA_003557565.1), as well as marine environments, indicating halotolerance within the family. Hydrogenotrophic sulfate reduction in another AHI spring, Lost Hammer Spring (24% salinity, − 5 °C), was attributed to a MAG in the uncultured BM506 genus in *Desulfobacterota*. This BM506 MAG was related at genus level (66% amino acid identity) to *Desulfurivibrionaceae* sp. GH16, suggesting that closely related novel taxa of halo- and psychrotolerant SRB are primarily responsible for sulfate reduction in the AHI springs. Hydrogen-dependent sulfate reduction was previously observed in microcosms of Lost Hammer Spring sediment down to − 20 °C [29], corroborating the hydrogenotrophic gene transcription detected in SRB from both springs and indicating that these SRB are highly adapted to cold down to the known temperature limits of microbial metabolic activity.

While the majority of sulfate reduction activity was attributed to *Desulfurivibrionaceae* sp. GH16, all six *Desulfobacterota* MAGs transcribed sulfur species reduction genes (Fig. 4). Three additional *Desulfobacterota* MAGs transcribed sulfate reduction genes, either to sulfide (GH12, GH14) or sulfite (GH13); both GH12 and GH13 also transcribed group 1a or group 1c [NiFe] hydrogenases involved in hydrogenotrophic respiration, supporting hydrogen-coupled sulfate reduction as a major metabolism in the spring (Fig. 4). As in GH16, all *Desulfobacterota* MAGs additionally transcribed N reduction genes and genes for reduction of multiple sulfur species, and all but one contained the Wood-Ljungdahl pathway for CO_2_ fixation (Fig. 4), indicating the GH spring supports a diversity of novel chemolithoautotrophic SRB utilizing S and N compounds as terminal electron acceptors.

Notably, transcription of sulfate reduction genes was also identified in MAGs from phyla not typically associated with sulfate reduction activity [57]. *Sphaerochaetales* sp. GH28 (*Spirochaetota*) transcribed genes for complete sulfate reduction to sulfide, as well as hydrogenotrophic group 1b and 1c [NiFe] hydrogenases and the Wood-Ljungdahl pathway, indicating similar metabolic activity as GH *Desulfobacterota*. *Spirochaetota* (formerly *Spirochaetes*) are typically chemoorganotrophs growing under a wide range of oxygen concentrations [69]; the phylum includes halophiles [70], and *Spirochaetota* have been identified in extreme cold hypersaline environments such as brines in ice-covered Lake Vida in Antarctica (− 13 °C) [71]. Sulfate reduction genes were only recently identified in this phylum, in the anoxic, sulfide-rich Zodletone Spring in Oklahoma, USA [72], and in groundwater in Tennessee, USA [73]. *Sphaerochaetales* sp. GH28 was novel at the family level in comparison to the GTDB database, and a 16S rRNA gene in the MAG (1539 bp) had 88% identity to its closest match in the SILVA database, suggesting this GH MAG represents a highly novel lineage of sulfate-reducing *Spirochaetota*.

Gene transcription for complete sulfate reduction to sulfide was also identified in UBA2268 sp. GH34, in the order *Kapabacteriales* and phylum *Bacteroidota*. In the GTDB database, this MAG was most closely related to a recently described ca. Kapabacteria from Yellowstone hot spring microbial mats representing the first sulfate-reducing bacterium from the *Bacteroidetes/Chlorobi* superphylum [74]. An additional sulfate-reducing ca. Kapabacteria was also recently reported in a deep subsurface aquifer [75]. Comparison of genomes by amino acid identity indicated that UBA2268 sp. GH34 was in the same family as both previously identified ca. Kapabacteria. All three *Kapabacteriales* SRB appear to be heterotrophic and contain genes involved in dissimilatory nitrite reduction (*nrfA*), denitrification (*nosZ* or *norB*), and polysulfide/thiosulfate reduction (*psrA/phsA*), as well as cytochrome c oxidases (*coxBAC* and/or *cydAB*), indicating similar metabolic function despite their presence in distinct and disparate environments of cold hypersaline GH, 60 °C oxygenic phototrophic microbial mats in non-saline hot springs in the USA and Japan, and 20 °C anaerobic non-saline subsurface aquifer waters in Western Siberia, Russia. Transcriptional studies of the Yellowstone hot spring ca. Kapabacteria suggested it may be facultatively aerobic and respire both sulfate and oxygen [74]; if so, this might give the UBA2268 sp. GH34 a unique niche in the upper portions of the GH sediment where trace oxygen is present compared to strictly anaerobic SRB.

While sulfate reduction genes were the most highly transcribed metabolic genes identified in the metatranscriptome, the most abundant *Desulfobacterota* present in the metagenome did not contain any sulfate reduction capabilities. *Desulfuromusa* sp. GH17 was the most abundant MAG in the metagenome (9.4% relative abundance) and the metatranscriptome, containing 20% of total relative transcript abundance (203,101.17 tpm) (Fig. 1b). The *Desulfuromusa* genus also comprised 14.4% of the 16S rRNA gene amplicon sequences, indicating significant abundance and activity of this taxon in the spring sediment (Fig. 1a). Previous 16S rRNA gene clone library and amplicon sequencing of the GH sediment also identified *Desulfuromusa* and *Desulfuromonadaceae* comprising up to 60% of detected *Desulfobacterota* [3, 32], demonstrating their abundance in GH over time. Previous comparison of cell-specific sulfur reduction rates with GH sulfur isotopic fractionation indicated that only ~ 60% of *Desulfurobacterota* in the GH sediment were engaged in active sulfate reduction [32], supporting the presence of significant populations of non-SRB *Desulfobacterota* such as *Desulfuromusa*.

Cultured *Desulfuromusa* are obligate anaerobes that typically oxidize or disproportionate organic C compounds and reduce a variety of electron acceptors including elemental sulfur, iron, manganese, nitrate, and fumarate [76, 77]. A psychrophilic *Desulfuromusa* has previously been cultured from cold Arctic marine sediments (− 2 °C) [77]. *Desulfuromusa* have also been identified by 16S rRNA gene amplicon sequencing and metagenomic sequencing in sulfidic anoxic marine environments such as hydrothermal vent fluids [78] as well as cold (− 1.8–7.5 °C) and saline (1–5%) Antarctic lakes [79].

Elemental sulfur (S^0^) has previously been identified in GH sediments, and it is a major component of the precipitate mineral crusts surrounding the springs [5]. Elemental sulfur is produced biotically in the spring outflow channels, where microbial streamers formed by *Thiomicrorhabdus* sp. oxidize sulfide to elemental sulfur [33, 80]. MAGs detected in this study (including *Thiomicrorhabdus* sp.) also transcribe sulfide oxidation genes (Fig. 4) indicating elemental sulfur is also produced biotically in the spring sediment, as discussed below. *Desulfuromusa* 16S rRNA gene clone sequences were identified within the *Thiomicrorhabdus* sp. microbial streamers, which contain S^0^ mineral structures [80]. *Desulfuromusa* were also more abundant in spring channel sediments compared to the primary outlet, indicating a potential positive association with *Thiomicrorhabdus* sp. streamers in the channels [32]. While elemental sulfur reduction by *Desulfuromusa* isolates has previously been described, no in-depth description of a *Desulfuromusa* genome has previously been published and the method of elemental sulfur reduction in this genus remains unclear. Two mechanisms of sulfur reduction have previously been characterized, utilizing either an [NiFe] hydrogenase and sulfur or polysulfide reductase (SreABCDE/PsrABC), or via an NADPH elemental sulfur oxidoreductase (NSR) [81, 82]. Potential homologs to these proteins were identified in *Desulfuromusa* sp. GH17 by BLAST using query proteins for NSR and NSR-like proteins from S^0^-reducers *Pyrococcus furiosus* and *Thermovibrio ammonificans* as well as Sre/Psr genes from a variety of bacterial taxa (complete output and list of query sequences in Table S13). *Desulfuromusa* sp. GH17 contained a homolog to *T. ammonoficians* NSR-like protein (31.6% identity, 152 bitscore) as well as weak homologs to *P. furiosus* NSR (22–24% identity, 52–56 bitscore). However, these homologs did not contain the conserved cysteine residue required for S^0^ reduction activity in some species [81]. A homolog to PsrB (34% identity, 70 bitscore) and a weak homolog to PsrA (22% identity, 97 bitscore) were also identified, although they were not located in the same operon as would be expected; additionally, no PsrC homolog was identified. All putative homologs had mapped transcripts. Thus, a mechanism for elemental sulfur reduction is possible in *Desulfuromusa* sp. GH17, potentially utilizing an NSR-like oxidoreductase; detected activity with an isolated strain would be necessary to confirm this activity.

**Fig. 5:**
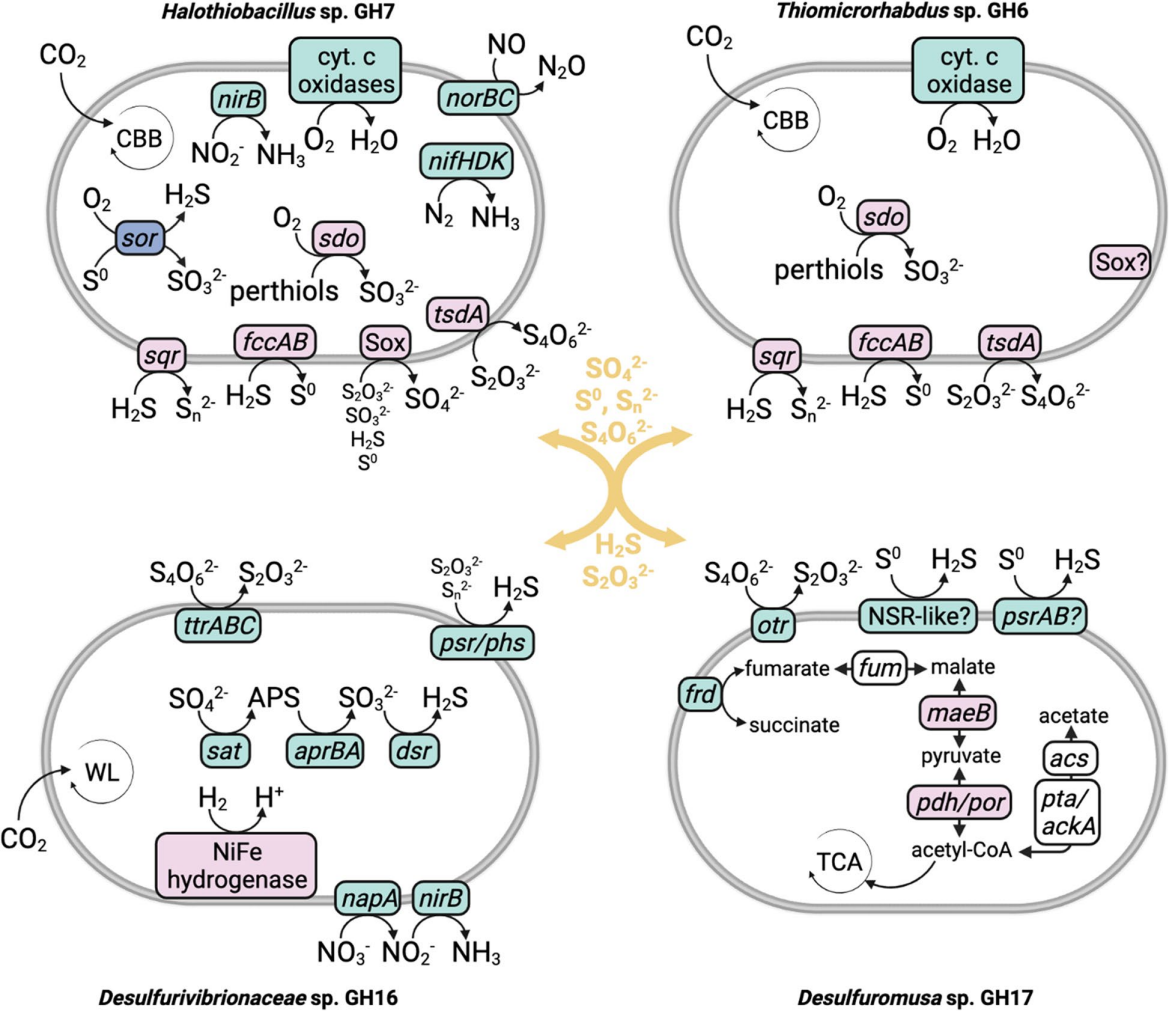
Genome content of key sulfur-cycling MAGs. Genes involved in oxidation reactions are in pink; reduction in green; disproportionation in blue. Yellow text indicates sulfur species putatively exchanged between S-oxidizing and S-reducing bacteria. Complete gene names and genome content are located in Table S10. Abbreviations are as follows: CBB, Calvin Benson Bassham cycle; NSR, NADH-dependent sulfur reductase; TCA, tricarboxylic acid cycle; WL, Wood-Ljungdahl pathway

The *Desulfuromusa* sp. GH17 also contains potential pathways for fumarate and malate disproportionation, fumarate reduction, and tetrathionate reduction, indicating possible alternate electron acceptors to sulfur, as well as acetogenic fermentation (Fig. 5a). Transporters for acetate, C4-dicarboxylates, and branched-chain amino acids were highly transcribed within the MAG (Table S14), indicating that it oxidizes or disproportionates organic carbon compounds as found in other *Desulfuromusa* spp.; it also appears to be obligately heterotrophic. However, the majority of relative transcript abundance in *Desulfuromusa* sp. GH17 was attributed to a single gene (Ga0534206_017155_28837_29236), accounting for 80.2% of relative transcript abundance in the MAG and 16% of relative transcript abundance in the entire metatranscriptome (162,861.6 tpm). This gene was classified as RNase P class A, a ubiquitous ribonuclease responsible for maturation of tRNAs and cleavage of a variety of other RNAs [83]. The next two most highly transcribed genes in the MAG were also non-coding RNAs classified as 6S/SsrS RNAs (26,427.2 and 1196.1 tpm), which act as transcriptional regulators of RNA polymerase. They are associated with switching from exponential to stationary growth phases, but have also been found to regulate large numbers of genes during the exponential phase [84]. It is not clear why these non-coding RNAs account for such a large proportion of *Desulfuromusa* sp. GH17 transcription. One possibility is that *Desulfuromusa* sp. GH17 modulates its metabolic strategy frequently based on substrate availability, requiring significant regulation of gene transcription. This could potentially result from availability of elemental sulfur and organic carbon compounds: while *Desulfuromusa* spp. are typically obligate anaerobes, elemental sulfur-producing microbes such as *Thiomicrorhabdus* sp. in GH are aerobic. Thus, they likely cannot exist in close proximity on a microscale in the sediment, potentially limiting access of *Desulfuromusa* sp. to biotic elemental sulfur. Organic carbon is also limiting in GH [30], potentially requiring changes in cellular activity, growth, or metabolic strategy based on availability of organic C. If *Desulfuromusa* sp. GH17 are indeed engaged in intensive gene regulation based on substrate availability, this may explain their significant abundance in the sediment compared to the chemolithoautotrophic *Desulfurobacterota* also present in GH, despite their reliance on limited organic C and inability to reduce non-limiting sulfate.

### Trace oxygen supports S-oxidizing Gammaproteobacteria

Oxidation of sulfur species was also widespread, with 15 MAGs (26%) containing S oxidation-related genes with mapped transcripts in the *Gamma*- and *Alphaproteobacteria*, *Campylobacterota*, *Bacteroidota*, and *Spirochaetota* (Fig. 4). S oxidation activity was linked with both aerobic and anaerobic metabolism: of the 15 MAGs, 11 transcribed N reduction genes and 11 transcribed terminal oxidases; 8 of these MAGs transcribed both N reduction genes and cytochrome c oxidases, indicating potential facultatively anaerobic microorganisms. Previous isolates from the GH spring sediment, including isolates containing *soxB* genes as markers of sulfur oxidation capability, were found to be predominantly facultative anaerobes, indicating this is likely advantageous in the anoxic spring sediment (0.04 ppm O_2_) [4]. All 11 MAGs transcribed high-affinity terminal oxidases (*cydAB* and/or *ccoNOPQ*), exclusively (4 MAGs) or in addition to low-affinity terminal oxidases (7 MAGs), indicating they are able to carry out respiration under low oxygen conditions. The low-affinity oxidases may also contribute to respiration even under anoxic conditions and could even be more favorable than high-affinity oxidases at microaerobic O_2_ concentrations due to more efficient ATP production, as found in some *Acidobacteria* [85]. Six of the 15 MAGs, from the *Gamma*- and *Alphaproteobacteria* and *Campylobacterota*, additionally contained pathways for CO_2_ fixation (CBB or rTCA cycles).

The Sox genes were the most highly transcribed S oxidation genes (353.9 tpm across 6 genes). The Sox complex oxidizes thiosulfate, sulfite, sulfide, elemental sulfur, and tetrathionate compounds, either completely to sulfate or, in the absence of *soxCD*, to elemental sulfur [86]. Sox genes were identified in 8 of the 15 S-oxidizing MAGs, from *Gammaproteobacteria*, *Alphaproteobacteria*, and *Campylobacterota*. Four MAGs contained genes for complete oxidation to sulfate and 3 MAGs contained genes for oxidation to sulfur (the remaining MAG, *Thiomicrorhabdus* sp. GH6, contained a partial Sox pathway with unclear functionality) (Fig. 4). Other S oxidation genes with high transcript abundances were *fccAB* (86.5 tpm) and *sqr* (103.6 tpm), which oxidize sulfide to elemental sulfur and polysulfide, respectively. Nearly all S oxidation gene transcription (96%) was attributed to *Gammaproteobacteria* (Fig. 3). Over half of this transcription was attributed to two MAGs, *Halothiobacillus* sp. GH7 (33% of total S oxidation gene transcription) and *Thiomicrorhabdus* sp. GH6 (24%).

*Thiomicrorhabdus* was abundant in the 16S rRNA gene amplicon sequences (4.8%) (Fig. 1a), and *Thiomicrorhabdus* sp. GH6 was the most abundant S-oxidizing MAG (3.5% relative abundance; Fig. 1b) in the shotgun metagenome. *Thiomicrorhabdus* spp. are characteristically chemolithoautotrophic S oxidizers and have previously been identified in cold saline environments including Arctic marine sediments [87] and Antarctic subglacial brines [88]. *Thiomicrorhabdus* (previously identified as *Thiomicrospira*) has previously been detected in abundance in the GH sediment [3], and, as noted, it forms microbial streamers in the spring channels where it aerobically and chemolithoautotrophically oxidizes sulfide to elemental sulfur [33]. Comparison of *Thiomicrorhabdus* sp. GH6 to the *Thiomicrorhabdus* sp. MAG recovered from GH streamers indicated that they were the same species (99.1% ANI). The genome and transcriptome of the streamer *Thiomicrorhabdus* MAG have previously been described in detail [33]; as expected, metabolic gene presence and transcription in the *Thiomicrorhabdus* sp. GH6 was largely identical. The most highly transcribed S-oxidizing genes in *Thiomicrorhabdus* sp. GH6 were the *fccAB* genes (85.7 tpm) involved in sulfide reduction to elemental sulfur, comprising nearly all (99%) of *fccAB* transcription in the metatranscriptome, corroborating its previously observed role in biotic elemental sulfur production in the spring. It additionally transcribes genes for sulfide oxidation to polysulfide (*sqr*, 26.4 tpm), thiosulfate oxidation (*tsdA*, 0.3 tpm), and sulfur dioxygenase (*sdo*, 1.2 tpm). Transcription of Sox genes was also detected (28.1 tpm). As in the streamer *Thiomicrorhabdus*, only a partial Sox complex (*soxBCD*) was identified in the MAG (Table S9). The *soxBCD* genes were also the only Sox genes identified in the streamer MAG, and while *soxXAY* genes classified as *Thiomicrorhabdus* were identified in unassembled reads, no *soxZ* was identified in the streamer metagenome. Thus, despite the *soxBCD* comprising a significant proportion of S oxidation gene transcription in *Thiomicrorhabdus* sp. GH6, the functionality of this complex remains unclear.

*Halothiobacillus* sp. GH7 had the highest transcript abundance of S oxidation genes overall (201.2 tpm), and among the highest total transcript abundance of the S-oxidizing MAGs (Fig. 1b). Cultured *Halothiobacillus* spp. are halotolerant, obligately aerobic, and chemolithoautotrophically oxidize S species [89–91], and members of this genus have previously been identified in sulfidic and hypersaline environments [89, 92]. The majority of transcript abundance of S oxidation genes by *Halothiobacillus* sp. GH7 was attributed to Sox genes (148.3 tpm), comprising 40% of total Sox transcript abundance. Homologs to all Sox genes except *soxX* were identified in the MAG (Table S9), indicating it likely utilizes the Sox complex for complete oxidation of S species to sulfate. It also transcribed a number of additional S oxidation genes, indicating it can utilize diverse S species including sulfide (*sqr*, 26.2 tpm), persulfides (*sdo*, 19.3 tpm), and thiosulfate (*tsdA*, 7.5 tpm). It is also the only MAG containing sulfur oxygenase/reductase (*sor,* 9.3 tpm), which aerobically disproportionates and oxygenates elemental sulfur to sulfide, sulfite, and thiosulfate in a non-energy-generating reaction as the first step in oxidation of the resulting products [93]. Nearly all *sor* transcript abundance in the metatranscriptome (97%) mapped to *Halothiobacillus* sp. GH7, indicating this may be a unique mechanism within the spring microbial community. It also transcribed all four cytochrome c oxidases and the CBB cycle for CO_2_ fixation (Fig. 4), indicating it is capable of aerobic, chemolithoautotrophic metabolism as in other *Halothiobacillus* spp. Additionally, the *Halothiobacillus* sp. GH7 transcribed genes involved in dissimilatory reduction of nitrite (*nirB*) and nitric oxide (*norBC*), as well as nitrogen fixation genes (*nifHDK*), suggesting it is potentially capable of anaerobic respiration. As noted, *Halothiobacillus* spp. are characteristically obligate aerobes, and *Halothiobacillus* found in environments with oxic-anoxic gradients such as marine haloclines have been restricted to oxic or micro-oxic zones [72, 92, 94]. However, *Halothiobacillus* in mine tailings containing an oxygen gradient in southern Ontario, Canada, were also found to transcribe *nirB*, *norBC*, and Sox genes under micro-oxic conditions, though the majority of Sox gene transcription was identified in oxic zones [95]. *Halothiobacillus* sp. GH7 N reduction gene transcripts were at low abundance (1.5 tpm) compared to terminal oxidases (54 tpm), indicating these *Halothiobacillus* spp. are primarily aerobic but may be capable of anaerobically respiring nitrogen species when oxygen is scarce. *Halothiobacillus* have been observed to produce nitrite from nitrate but not grow under anaerobic conditions [89], indicating that anaerobic respiration may supplement aerobic respiration but not enable growth.

### Chemolithoautotrophy rather than photoautotrophy likely sustains primary production

Due to the high latitude of the GH spring, it experiences seasonal periods of both constant illumination (~ May to July) and complete darkness (~ October to February). No evidence of photoautotrophy has previously been identified in the GH spring source, even during summer illumination [4]. Seventeen of the GH MAGs contained CO_2_ fixation pathways, accounting for 20% of total relative transcript abundance; the majority, accounting for 17% of total relative transcript abundance, were S-cycling microorganisms in the *Desulfobacterota*, *Gamma*- and *Alphaproteobacteria*, *Spirochaetota*, and *Campylobacterota*. No MAGs contained genes indicating photosynthetic capability although transcripts mapped to unbinned photosystem I (*psaAB*; 12.5 tpm) and photosystem II (*psbAB*; 351.6 tpm) marker genes in the metagenome. Each photosynthesis-related gene with mapped transcripts was present in a single copy in the metagenome, and comparison of all four proteins to the NCBI nr database indicated they are most similar to chloroplast in photosynthetic eukaryotes, with *psaAB* most closely related to *Nitzschia anatoliensis* chloroplast genes (~ 99% identity) and *psbAB* to *Durinskia baltica* (> 99% identity). *Nitzschia* spp. diatoms include psychrophilic and halotolerant species found in Arctic sea ice [96], while *Durinskia baltica* are dinoflagellates inhabiting freshwater and marine environments [97]. Both *Nitzschia* and *Durinskia* 18S rRNA gene sequences were identified in the metagenome at low abundance (< 0.03%). The low abundance of these eukaryotes, as well as the low abundance of *Eukaryota* (0.3% of metagenomic reads, 0.6% of metatranscriptomic reads) and *Cyanobacteria* (< 0.4% in both the metagenome reads and 16S rRNA gene amplicon sequences, 0.1% of metatranscriptomic reads), indicates that while there is some amount of photoautotrophy occurring in the spring sediment during summer illumination, it is evidently insufficient to sustain large populations of photoautotrophic eukaryotes or bacteria. A low level of transcription (0.12 tpm) was detected for *pufM*, involved in anoxygenic photosynthesis. The *pufM* gene with mapped transcripts was most homologous (98% identity) to *pufM* from *Roseovarius tolerans* (RefSeq ID WP_076787673) when classified by JGI Phylo_dist. *Roseovarius* is a member of the *Roseobacter* clade, which is metabolically diverse and includes chemoheterotrophs, lithoautotrophs, photoheterotrophs, and mixotrophs [98, 99]. Two *Roseobacter* clade spp. (*Roseobacter* sp. and *Loktanella* sp.), as well as an *Alkalibacterium* sp. (typical chemoheterotrophs) [100], containing *pufM* genes were additionally previously isolated from the spring sediment, indicating some level of anoxygenic photosynthesis occurs. While the functional diversity of these clades makes their potential contribution to spring primary production unclear, the low level of transcription suggests it is not a major process in the sediments. Thus, this study indicates that a low level of photosynthesis, including photoautotrophy, occurs in the spring sediment during summer illumination. However, the low abundance and activity of these microbes suggests their contribution to primary production is limited and potentially seasonal, with chemolithoautotrophic sulfur-cycling bacteria likely sustaining the majority of primary production year-round.

Sulfur-based chemolithoautotrophy as a main driver of microbial primary production is common in aphotic and light-limited S-rich environments, such as deep-sea marine environments and cave sulfidic springs [21, 101]. By contrast, predominantly chemolithoautotrophic systems are rare in illuminated surficial environments such as GH, including other Arctic environments that experience seasonal darkness such as sea ice where algae are the primary producers [102]. Thus, other parameters of the GH spring may be inhibitory to photoautotrophic microorganisms. High sulfide concentrations, including at the level found in GH, can inhibit oxygenic phototrophs, though some *Cyanobacteria* can overcome this inhibition by switching to anoxygenic photosynthesis [103]. Sulfide can also inhibit some anaerobic anoxygenic phototrophs such as purple sulfur bacteria, and the low oxygen availability may additionally limit the activity of aerobic anoxygenic phototrophs [104]. Similar S-based chemolithoautrophy has also been observed at the Colour Peak and Lost Hammer AHI springs [6, 33], as well as High Arctic glacial sulfidic springs [22, 105], indicating that it occurs where sulfur is present in abundance to power chemosynthetic life.

It should be noted that this does not exclude additional photoautotrophy in the overlying spring waters (10^4^ cells/mL) [4], which were not examined in this study. While light penetration would likely restrict phototrophs to the top layer of sediment, previous studies also suggest limited potential for photoautotrophy in the spring waters. Measurements of CO_2_ uptake in GH spring waters found higher uptake in dark incubations compared to light-exposed incubations, indicating potential chemolithoautotrophic activity. Additionally, 16S rRNA gene sequencing of the nearby AHI Colour Peak spring water (5 °C, 16% salinity) found it to be dominated by sulfide-oxidizing, chemolithoautotrophic *Thiomicrospira* and did not detect any phototrophic taxa [34]. However, 18S rRNA gene and metagenomic sequencing of the spring water is required to corroborate these results. Additionally, it is unclear why CO_2_ fixation might be higher under darkness; this may represent effects of the microcosm incubation or potential inhibitory interactions between photoautotrophs and chemolithoautotrophs. Thus, while the spring sediments appear to be predominantly chemolithoautotrophic, additional analyses including the spring waters is needed to fully describe the GH spring system.

**Fig. 6:**
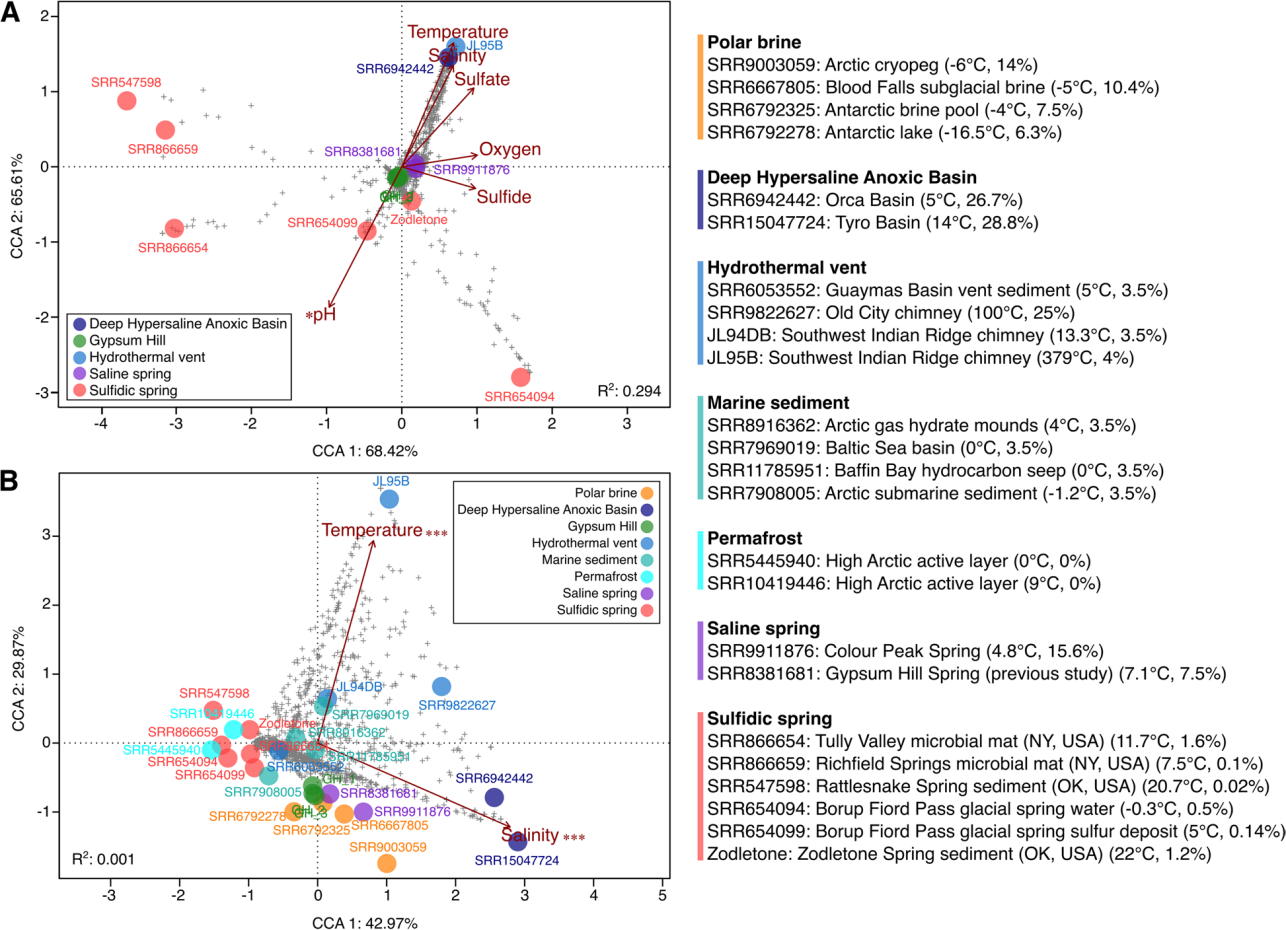
**A** Plot of canonical correspondence analysis (CCA) of 16S rRNA gene sequencing from GH and cold, saline, and sulfur-rich environments relating environmental variables to taxonomic composition of the samples (ANOVA F = 1.46 and *p*-value = 0.003 **). Circles represent total samples, “ + ” indicates individual taxa, “*” represents statistically significant environmental variables of *p*-value ≤ 0.05. Sample metadata can be found in Table S4. **B** Plot of canonical correspondence analysis (CCA) of 16S rRNA gene sequencing from GH and additional comparable environments relating temperature and salinity to taxonomic composition of the samples (ANOVA F = 1.001 and *p*-value = 0.476). Circles represent total samples, “ + ” indicates individual taxa, “***” represents statistically significant environmental variables of *p*-value ≤ 0.001. Sample metadata can be found in Table S4

To contextualize the GH spring community, the 16S rRNA gene amplicon sequences were compared to sequences from 24 hypersaline, cold, and/or S-rich environments. Of the 24 datasets, a subset of 13 with sufficient metadata (including the GH replicates from this study) were selected for canonical correspondence analysis (CCA) to relate microbial community composition to environmental variables, though estimates were still required for some parameters (details in Table S4). A large proportion of sample variance was accounted for by the environmental variables (axis 1: 65.61% and axis 2: 68.42%, Fig. 6a), though only pH was a significant factor individually based on ANOVA (*r* ^2^ = 0.581, *p*-value = 0.028 *). The GH data from this study clustered relatively centrally without strong associations with environmental factors compared to very extreme samples from hydrothermal vents (JL95B; 379 °C, 3.42 pH) [106] and Deep Hypersaline Anoxic Basins (DHABs) (SRR6942442; 26.7% salinity) [107]. The GH data clustered most closely with saline spring samples representing previous sequencing of the GH spring sediment (SRR8381681) [32] and another AHI spring, Colour Peak (SRR9911876; 5 °C, 16% salinity) [6]. These saline springs were relatively correlated with low oxygen concentration and moderate hypersalinity in comparison with the highly extreme samples as well as with the sulfidic springs, which contain a range of environmental conditions but are characteristically non-saline. Variance in all 27 samples was also analyzed by CCA (Fig. 6b), though only temperature and salinity could be used as environmental factors due to lack of reported metadata. Despite this, a large proportion of sample variance was again explained by the environmental variables (axis 1: 42.97% and axis 2: 29.33%), with the very extreme hydrothermal vents and DHABs once again strongly correlated with temperature and salinity based on ANOVA (*r* ^2^ = 1, *p*-value = 0.001 ***). Comparatively, the GH samples and AHI saline springs most closely clustered with cold brines including Arctic cryopegs and Antarctic lake and subglacial brines, and were correlated with low temperature and moderate hypersalinity. A Spearman correlation of the most abundant taxa (top 50) present in the data sets showed the sulfur-cycling taxa found in GH (*Thiomicrorhabdus, Desulfuromusa, Desulfobacteraceae*) were positively correlated with salinity and sulfate and negatively correlated with oxygen (Figure S8), and both *Thiomicrorhabdus* and *Desulfobacteraceae* were also negatively correlated with temperature. An NMDS ordination of a weighted Bray–Curtis dissimilatory matrix for all 27 samples was also plotted to compare the differences between samples based on sample composition (without environmental factors) (Figure S9). Samples broadly clustered by category, reflecting the influence of environmental factors, with the GH and AHI saline springs clustering most closely with some brine samples as well as some sulfidic springs, including those at Borup Fjord in the Canadian Arctic (< 5 °C, < 0.5% salinity; SRR654094 and SRR654099) [22] and Zodletone Spring in Oklahoma, USA (22 °C, 1% salinity; “Zodletone”) [66].

### Gypsum Hill as a Mars analog

**Fig. 7:**
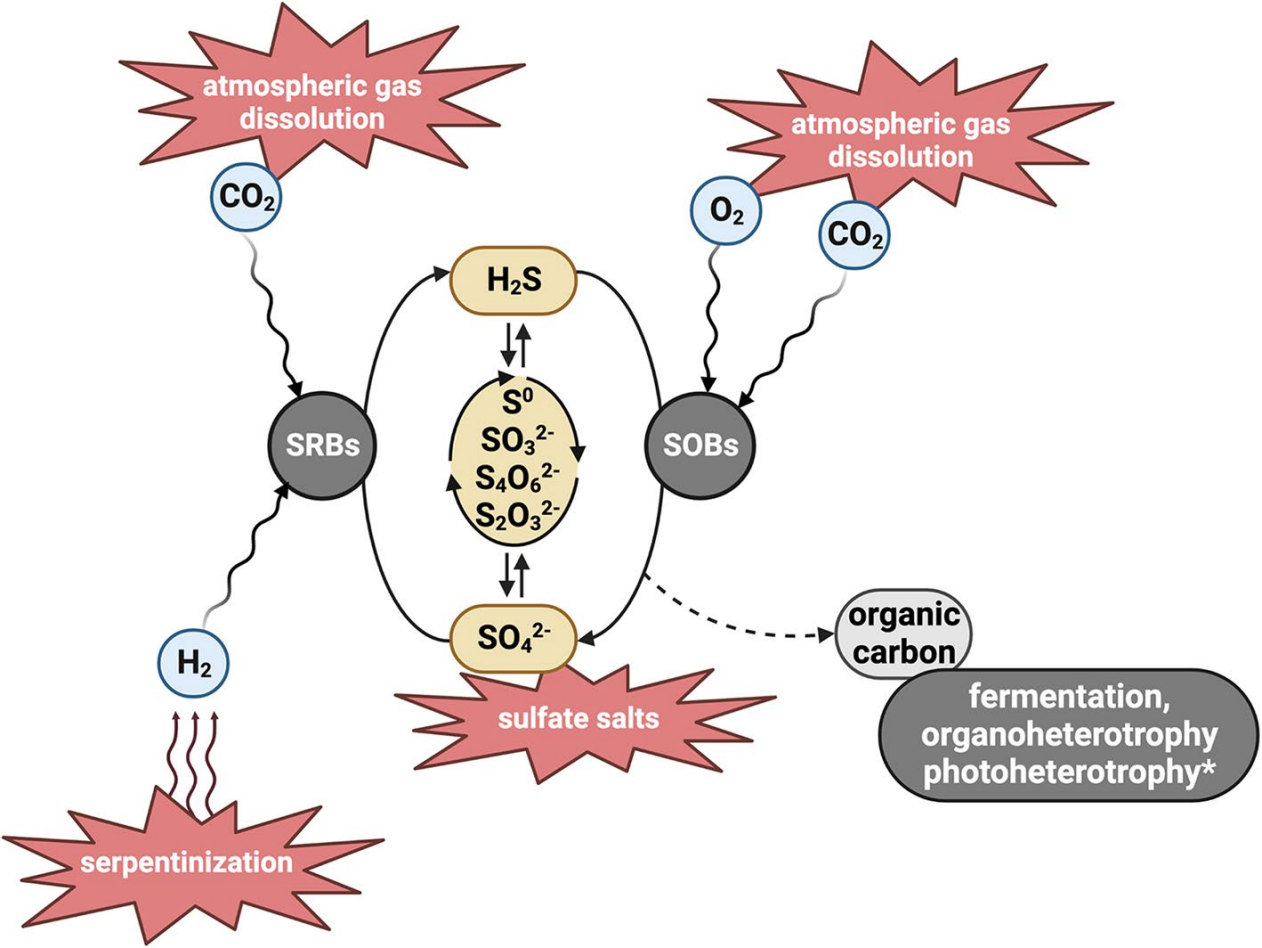
Model for a hypothetical sulfur-cycling microbial community similar to the GH microbial community in a theoretical Martian environment such as subsurface sulfate brines. Putative Martian sources of energy and carbon are indicated in red. SRBs refer to S-reducing bacteria and SOBs refers to S-oxidizing bacteria. The asterisk indicates phototrophic metabolism, which would be relevant only in very near-surface or surface environments on ancient Mars

Sulfate is abundant on Mars, including in putative cold, briny waters in the present subsurface and past surface of Mars. The sulfate-rich, cold, hypersaline, anoxic GH spring is an excellent analog site for identifying and constraining the microbial community and metabolisms that might be present in these environments (Fig. 7). We identified active and abundant H_2_-oxidizing, sulfate-reducing, chemolithoautotrophic *Desulfobacterota* as the likely predominant drivers of microbial primary production in the springs, utilizing abundant sulfate supplied from the subsurface. While hydrogen has not been detected in the near-surface Martian atmosphere, H_2_ is postulated to be present in the subsurface as a result of serpentinization reactions [108], where co-localization with sulfate-rich brines could create similar conditions for supporting SRBs to those found in GH.

While the majority of sulfur on the Martian surface is in the form of sulfate, sulfides have been detected in Gale Crater [109]. Additionally, jarosite also detected in Gale Crater may have formed from oxidation of Fe-sulfides [110], suggesting the presence of both oxidized and reduced sulfur on ancient Mars with some reduced sulfur persisting to the present. Sulfide and other reduced sulfur species produced by SRBs in GH support chemolithoautotrophic S-oxidizing bacteria. The majority of in situ activity was attributed to aerobic *Gammaproteobacteria* despite the highly reducing (− 430 mV) and oxygen-limited (~ 1.25 µM O_2_) conditions in the GH sediment, the relative availability of anaerobic electron acceptors such as nitrate (~ 6.5 µM), and the presence of anaerobic and facultatively anaerobic SOBs. However, even nanomolar concentrations of oxygen can sustain aerobic metabolisms due to the energetic favorability of O_2_ as an electron acceptor [111]. The relatively high energy yield of aerobic metabolisms is also favorable in hypersaline environments due to the energy expense of osmotic adaptations [112]. Oxygen is present in the Martian atmosphere at ~ 3 nM [113], and thermodynamic modeling of putative Martian near-surface perchlorate and sulfate brines suggested O_2_ could concentrate in these brines up to 2 µM, enabling aerobic respiration [114]. Molecular oxygen could also be produced through microbial metabolisms such as chlorite dismutation independent of photosynthesis and support significant populations of aerobes, as found in ancient groundwaters on Earth [115]. Our results highlight the potential importance of even trace oxygen concentrations to microbial metabolism in Martian environments. Active aerobic, facultatively aerobic, and anaerobic S-cycling bacteria were identified in situ in the GH spring sediments, indicating these oxygen-limited environments on Mars could support diverse S-linked metabolisms and microorganisms.

Genomes and transcripts of SRBs and SOBs in GH indicated redox of multiple S species in addition to sulfate and sulfide, and potential exchange of S intermediates between oxidizers and reducers (Fig. 5). Measurement of sulfur isotopic fractionation is one proposed method for identification of microbial sulfur metabolism on Mars [7]; NASA’s Curiosity rover has measured large variations in fractionations of sulfur isotopes in Gale Crater sediments [116]. Oxidation and disproportionation redox processes in addition to sulfate reduction increase S isotope fractionations, as can hypersaline stress, low temperatures, and limited available e-donors (organic C, H_2_) and acceptors (O_2_) as in GH and in putative habitats on Mars [7, 117]. Analog environments like GH can thereby inform interpretation of isotopic measurements on Mars and help distinguish biotic from abiotic signatures [7]. Previous sulfur isotope measurements in GH sediment are consistent with sulfate reduction without disproportionation and oxidative processes [33]; our results demonstrate that this does not rule out the presence of these metabolisms or reduction of additional sulfur species like elemental sulfur. This is relevant to the current Mars Science Laboratory mission (Curiosity rover) measuring sulfur isotope fractionations in Gale Crater [116], and for potential future sample return from the Perseverance rover which will allow for a larger suite of isotopic and other analyses. Notably, Perseverance recently cached sedimentary rock samples rich in salts and organic carbon [118, 119] formed during evaporation of the Jezero lake, where sulfates [120] and products of potential ancient serpentinization reactions [121] have been detected, representing an exciting candidate for future biosignature detection and demonstrating the importance of cold hypersaline sediment analog environments such as GH to current and future biosignature detection efforts.

Our results demonstrate that sulfur-based chemolithoautotrophy in GH supports additional metabolisms including chemoorganotrophs, heterotrophs, and fermenters (Fig. 4), including highly active and abundant microorganisms such as *Desulfuromusa*. This has implications for putative metabolisms that could exist or have existed in similar environments on Mars and their associated biosignatures [6]. For example, the potential for morphological, mineralogical, or isotopic biosignatures in sulfate-rich brines additional to those associated with SRB such as isotope fractionations associated with organoheterotrophy or iron and nitrogen redox; accumulations of trace element cofactors such as copper in cytochrome c oxidases; or deposition of Se and elemental sulfur by sulfide-oxidizers [122].

## Conclusions

Abundant sulfate in Gypsum Hill Spring supports an active microbial community dominated by sulfur-cycling metabolisms. A complete sulfur cycle through sulfate and sulfide, including cycling of sulfur intermediate species, supports a diversity of S-reducing and S-oxidizing microorganisms. The majority of these microorganisms demonstrate putative metabolic flexibility (oxidation and reduction of multiple S species, use of both S and N electron acceptors, facultative anaerobes), potentially in response to low energy availability (e.g., limited organic carbon and oxygen) in the spring. Our results demonstrate the potential for anoxic, cold, sulfate-rich brines to support microbial life on Mars, with implications for biosignature detection in these past/current habitats.

### Supplementary Information

**Additional file 1: Figure S1.**a. Photograph of GH-4 primary outlet and downstream channels (July 2019). A fine layer of gypsum coats the area around the springs. b. Location of the Gypsum Hill springs on Axel Heiberg Island, Nunavut, Canada (indicated with red dot). Map generated in QGIS with the Natural Earth dataset. c. Photograph of the Gypsum Hill springs area in which GH-4 is located. Photos: E. Magnuson. **Figure S2. **Phylogenetic tree of DsrAB sequences. **Figure S3. **Phylogenetic tree of DsrA sequences. **Figure S4.** Phylogenetic tree of DsrB sequences. **Figure S5**. Phylogenetic trees of DsrA and DsrB sequences. **Figure S6.** Relative abundance of reads in the metagenome and metatranscriptome classified by Kaiju using the NCBI non-redundant database (nr_euk). Relative abundance was averaged between replicates for both the metagenome and metatranscriptome. **Figure S7.** Level of taxonomic novelty of ASVs (2,885 ASVs in total). **Figure S8.** Spearman’s rank correlation of the top 50 most abundant taxa in the subset of thirteen 16S rRNA gene sequencing data sets with environmental parameters. Metadata for this plot is located in Table S4. **Figure S9.** NMDS plot with Bray Curtis dissimilarity matrix for 16S rRNA gene amplicon sequences from GH and comparable environments. Metadata for this plot is located in Table S4. **Table S1. **Physical and chemical parameters in GH-4. **Table S2. **Sequencing library statistics. **Table S3.** Metagenome co-assembly statistics. **Table S4. **Metadata for amplicon metagenome libraries used in beta diversity analysis. **Table S5. **Taxonomic count table used for beta diversity analysis. **Table S6. **List of contigs in each MAG. **Table S7. **MAG supplemental information. **Table S8. **Taxonomic classification of genes of interest with mapped transcripts. **Table S9. **Gene content of MAGs. **Table S10. **Relative expression of genes of interest in MAGs. **Table S11. **Total tpm per genome feature product ID. **Table S12. **Gene counts and relative expression of genes of interest in the metagenome. **Table S13. **Complete BLAST output of elemental sulfur reduction proteins queried against *Desulfuromusa *sp. GH17. **Table S14. **Relative expression of all genes.

## Data Availability

All sequencing data was deposited in NCBI under BioProject PRJNA915120. Metagenome annotation is available under Analysis Project ID Ga0534206 in the Joint Genome Institute IMG/M system. A list of MAG contigs is supplied in Table S6 so IMG metagenome annotation can be attributed to MAGs.

## Abbreviations

GH: Gypsum Hill
MAGs: Metagenome-assembled genomes
AHI: Axel Heiberg Island
ASV: Amplicon sequence variant
NMDS: Non-metric Multidimensional Scaling
CCA: Canonical Correspondence Analysis
AAI: Amino acid identity
ANI: Average Nucleotide Identity
SRB: Sulfate-reducing bacteria
SOB: Sulfur-oxidizing bacteria
DHAB: Deep Hypersaline Anoxic Basin
