# Supplementary material for: Sulfur-cycling chemolithoautotrophic microbial community dominates a cold, anoxic, hypersaline Arctic spring
Source: Microbiome. 2023 Sep 11;11:203. doi: 10.1186/s40168-023-01628-5 (PMC10494364; doi:10.1186/s40168-023-01628-5)
Supplement: Supplementary file 2 — Additional file 1: Figure S1.a. Photograph of GH-4 primary outlet and downstream channels (July 2019). A fine layer of gypsum coats the area around the springs. b. Location of the Gypsum Hill springs on Axel Heiberg Island, Nunavut, Canada (indicated with red dot). Map generated in QGIS with the Natural Earth dataset. c. Photograph of the Gypsum Hill springs area in which GH-4 is located. Photos: E. Magnuson. Figure S2. Phylogenetic tree of DsrAB sequences. Figure S3. Phylogenetic tree of DsrA sequences. Figure S4. Phylogenetic tree of DsrB sequences. Figure S5. Phylogenetic trees of DsrA and DsrB sequences. Figure S6. Relative abundance of reads in the metagenome and metatranscriptome classified by Kaiju using the NCBI non-redundant database (nr_euk). Relative abundance was averaged between replicates for both the metagenome and metatranscriptome. Figure S7. Level of taxonomic novelty of ASVs (2,885 ASVs in total). Figure S8. Spearman’s rank correlation of the top 50 most abundant taxa in the subset of thirteen 16S rRNA gene sequencing data sets with environmental parameters. Metadata for this plot is located in Table S4. Figure S9. NMDS plot with Bray Curtis dissimilarity matrix for 16S rRNA gene amplicon sequences from GH and comparable environments. Metadata for this plot is located in Table S4. Table S1. Physical and chemical parameters in GH-4. Table S2. Sequencing library statistics. Table S3. Metagenome co-assembly statistics. Table S4. Metadata for amplicon metagenome libraries used in beta diversity analysis. Table S5. Taxonomic count table used for beta diversity analysis. Table S6. List of contigs in each MAG. Table S7. MAG supplemental information. Table S8. Taxonomic classification of genes of interest with mapped transcripts. Table S9. Gene content of MAGs. Table S10. Relative expression of genes of interest in MAGs. Table S11. Total tpm per genome feature product ID. Table S12. Gene counts and relative expression of genes of interest in the metagenom [file 40168_2023_1628_MOESM1_ESM.zip › Magnuson2023_FiguresS1-S9_TablesS2-S3.docx]

**Supplementary information for “Sulfur-cycling chemolithoautotrophic microbial community dominates a cold, anoxic, hypersaline Arctic spring”, Magnuson, E., Altshuler, I., Freyria, N.J, Leveille, R.J., Whyte, L.G.**

**
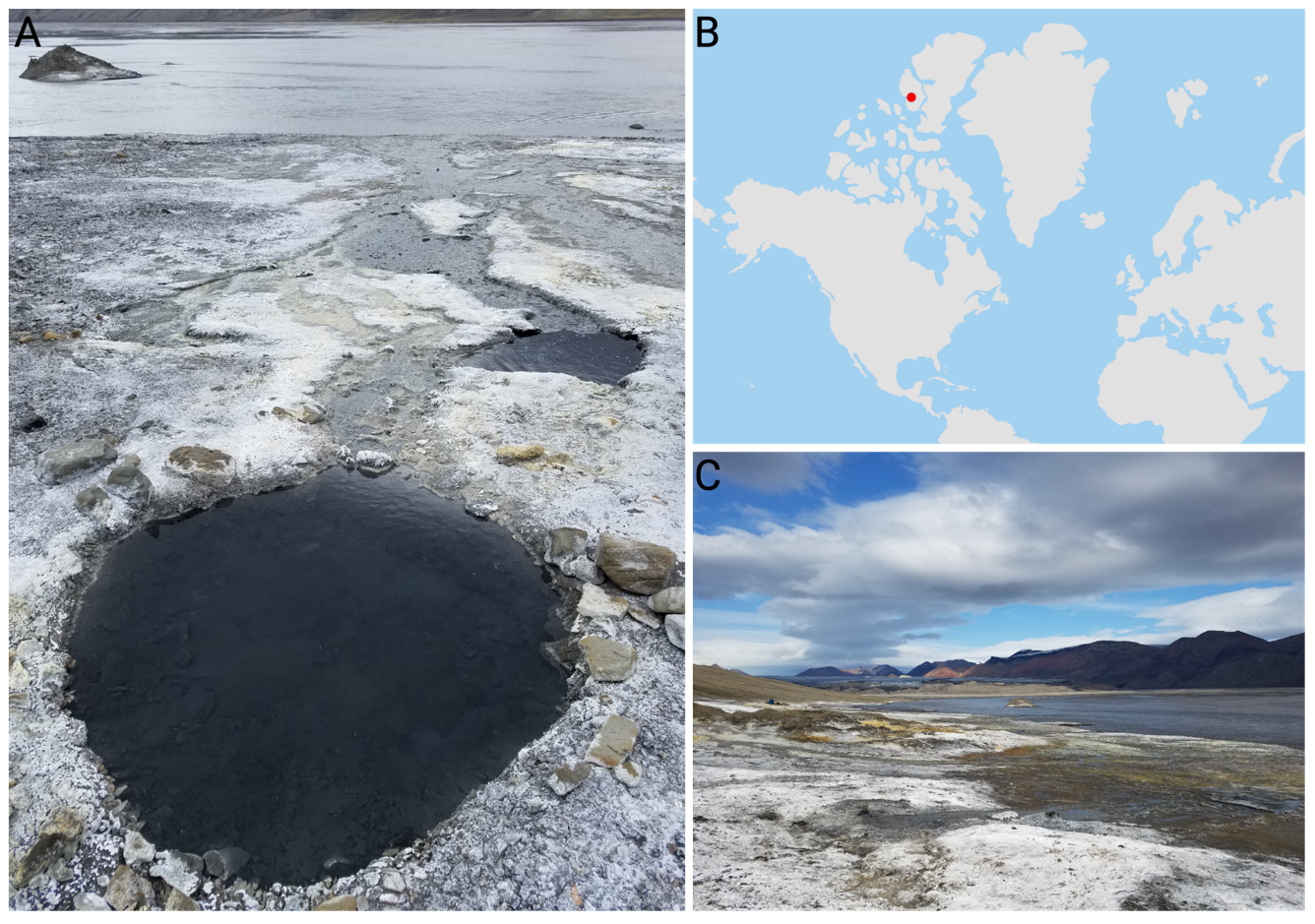
**

**Figure S1.** **a.** Photograph of GH-4 primary outlet and downstream channels (July 2019). A fine layer of gypsum coats the area around the springs. **b.** Location of the Gypsum Hill springs on Axel Heiberg Island, Nunavut, Canada (indicated with red dot). Map generated in QGIS with the Natural Earth dataset. **c.** Photograph of the Gypsum Hill springs area in which GH-4 is located. Photos: E. Magnuson.


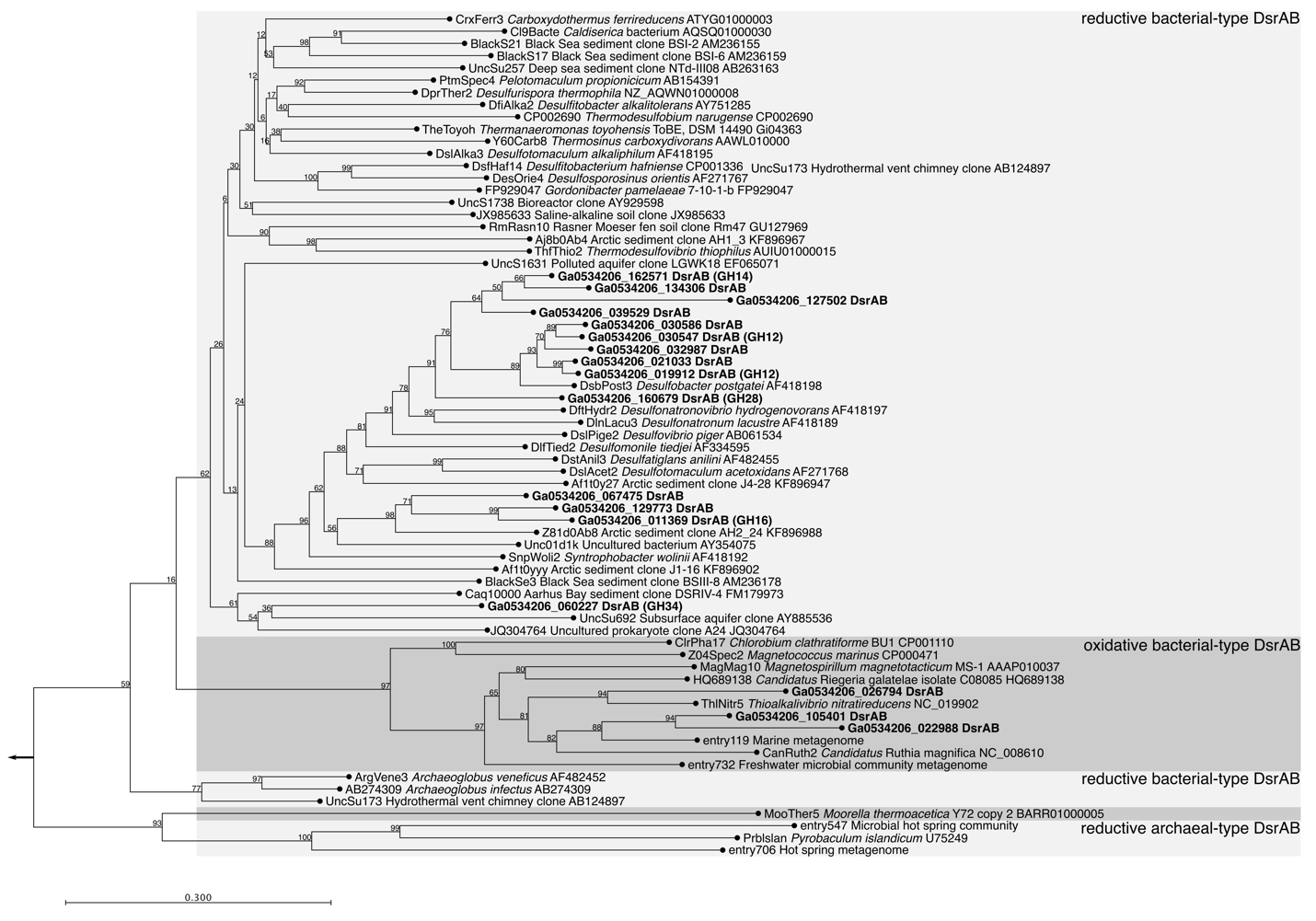


**Figure S2.** Phylogenetic tree of DsrAB sequences. The tree was constructed in CLC Genomics Workbench with 1000 bootstraps and WAG substitution model using reference sequences from Müller *et al*. (2015). Sequence names include the contig IDs and MAG ID where applicable. A sequence from eggNOG group COG2221 (*Campylobacter ureolyticus* JFJK01000015_gene476) was used as an outgroup (direction indicated by arrow).


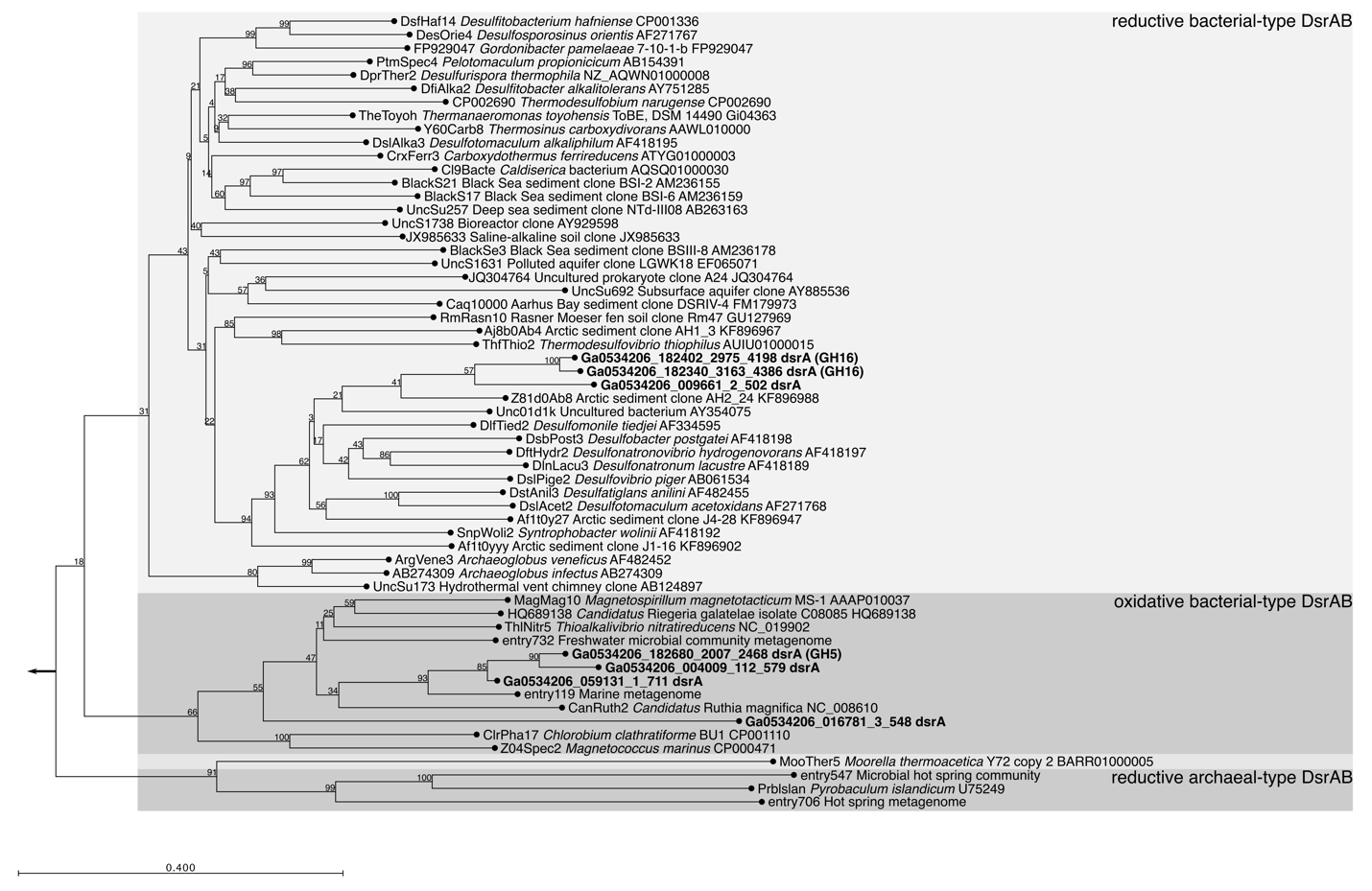


**Figure S3.** Phylogenetic tree of DsrA sequences. The tree was constructed in CLC Genomics Workbench with 1000 bootstraps and WAG substitution model using reference sequences from Müller *et al*. (2015). Sequence names include the contig IDs and MAG ID where applicable. A sequence from eggNOG group COG2221 (*Campylobacter ureolyticus* JFJK01000015_gene476) was used as an outgroup (direction indicated by arrow).


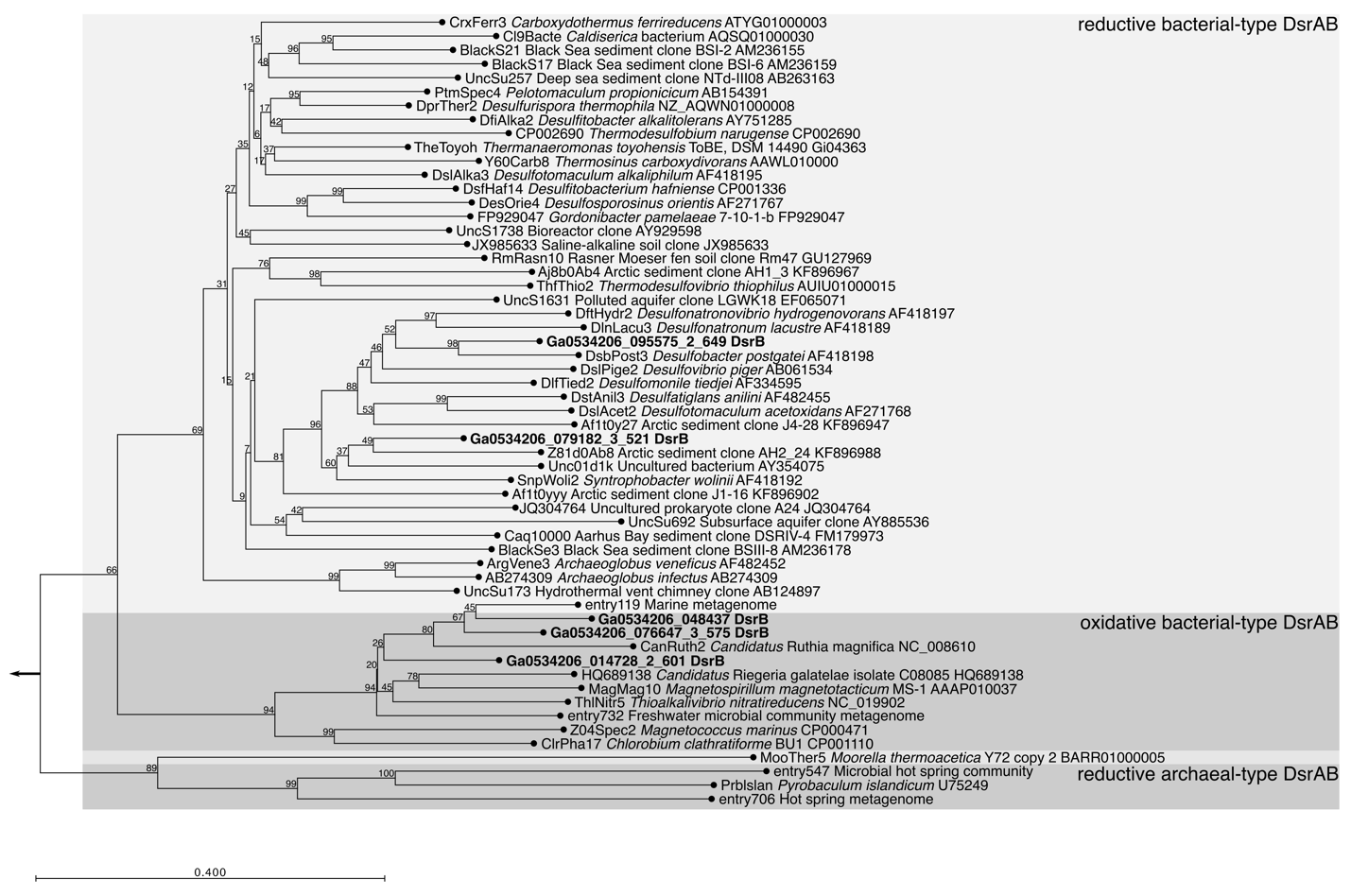


**Figure S4.** Phylogenetic tree of DsrB sequences. The tree was constructed in CLC Genomics Workbench with 1000 bootstraps and WAG substitution model using reference sequences from Müller *et al*. (2015). Sequence names include the contig IDs and MAG ID where applicable. A sequence from eggNOG group COG2221 (*Campylobacter ureolyticus* JFJK01000015_gene476) was used as an outgroup (direction indicated by arrow).


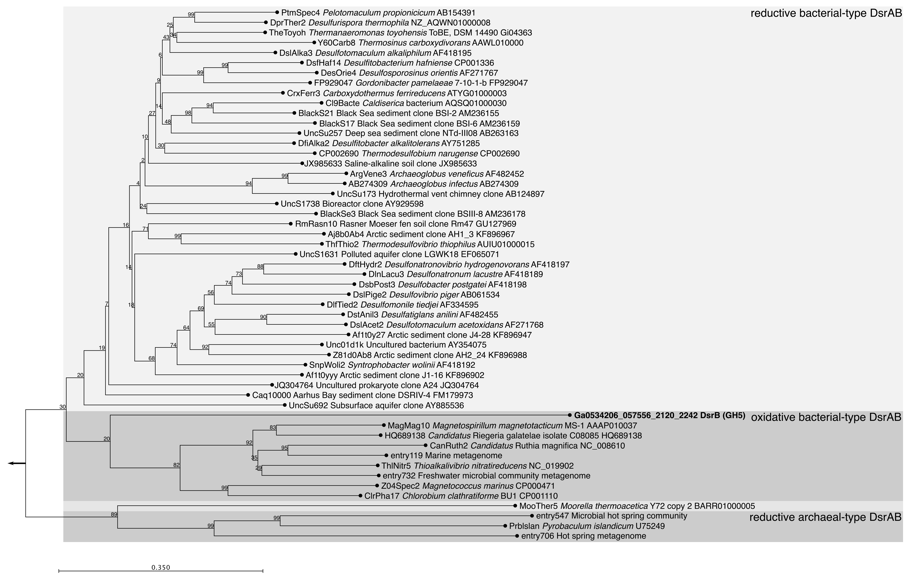


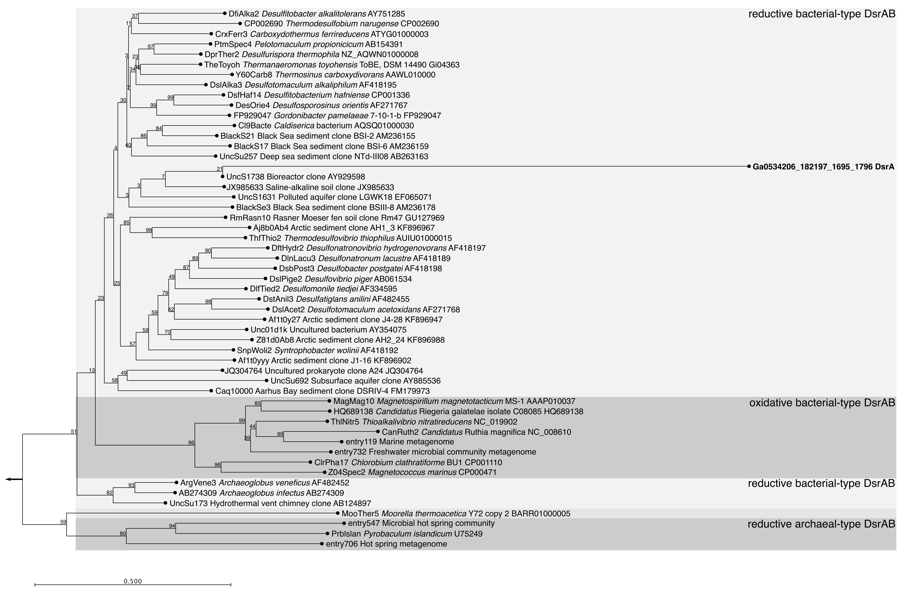


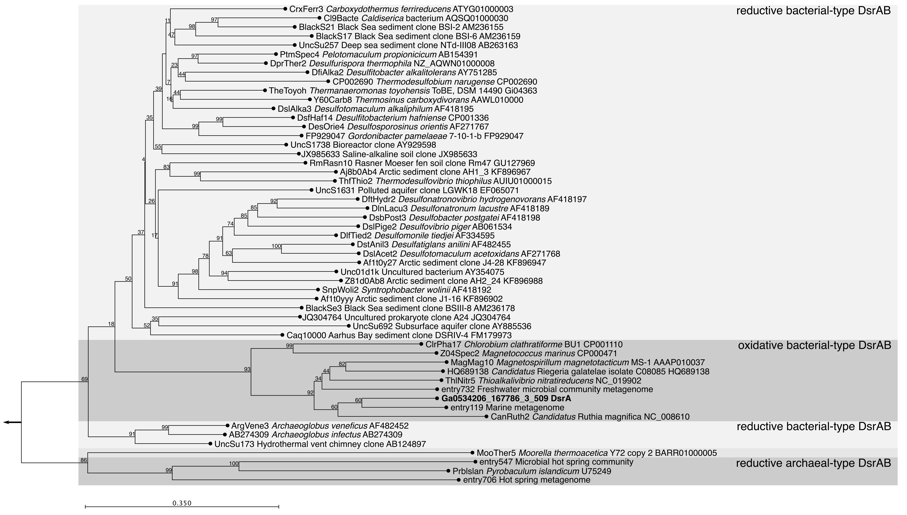


**Figure S5.** Phylogenetic trees of DsrA and DsrB sequences. These sequences were aligned separately due to lack of clustering when included with other DsrAB sequences as in Figures S2-S4. The trees were constructed in CLC Genomics Workbench with 1000 bootstraps and WAG substitution model using reference sequences from Müller *et al*. (2015). Sequence names include the contig IDs and MAG ID where applicable. A sequence from eggNOG group COG2221 (*Campylobacter ureolyticus* JFJK01000015_gene476) was used as an outgroup (direction indicated by arrow).


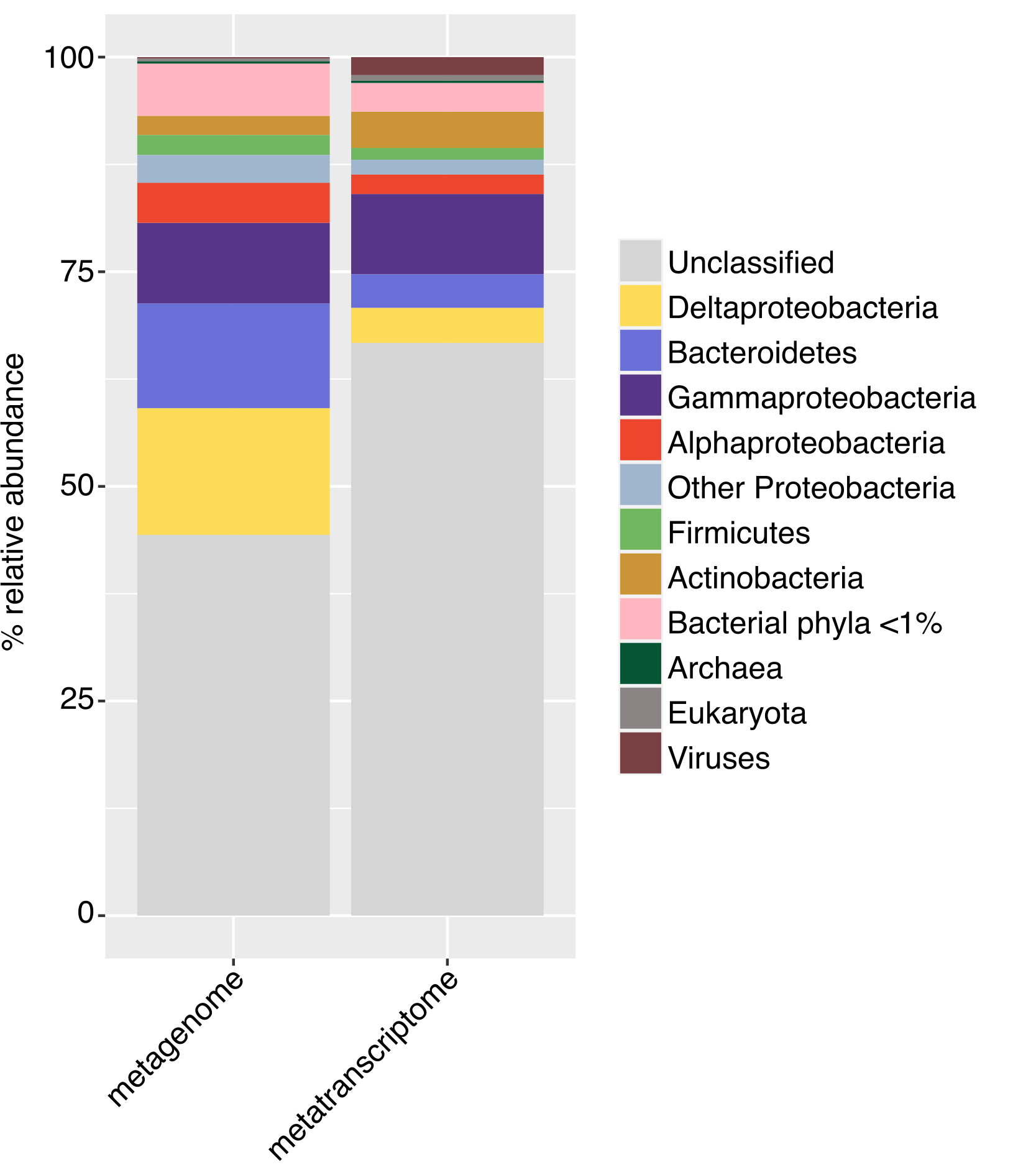


**Figure S6.** Relative abundance of reads in the metagenome and metatranscriptome classified by Kaiju using the NCBI non-redundant database (nr_euk). Relative abundance was averaged between replicates for both the metagenome and metatranscriptome.


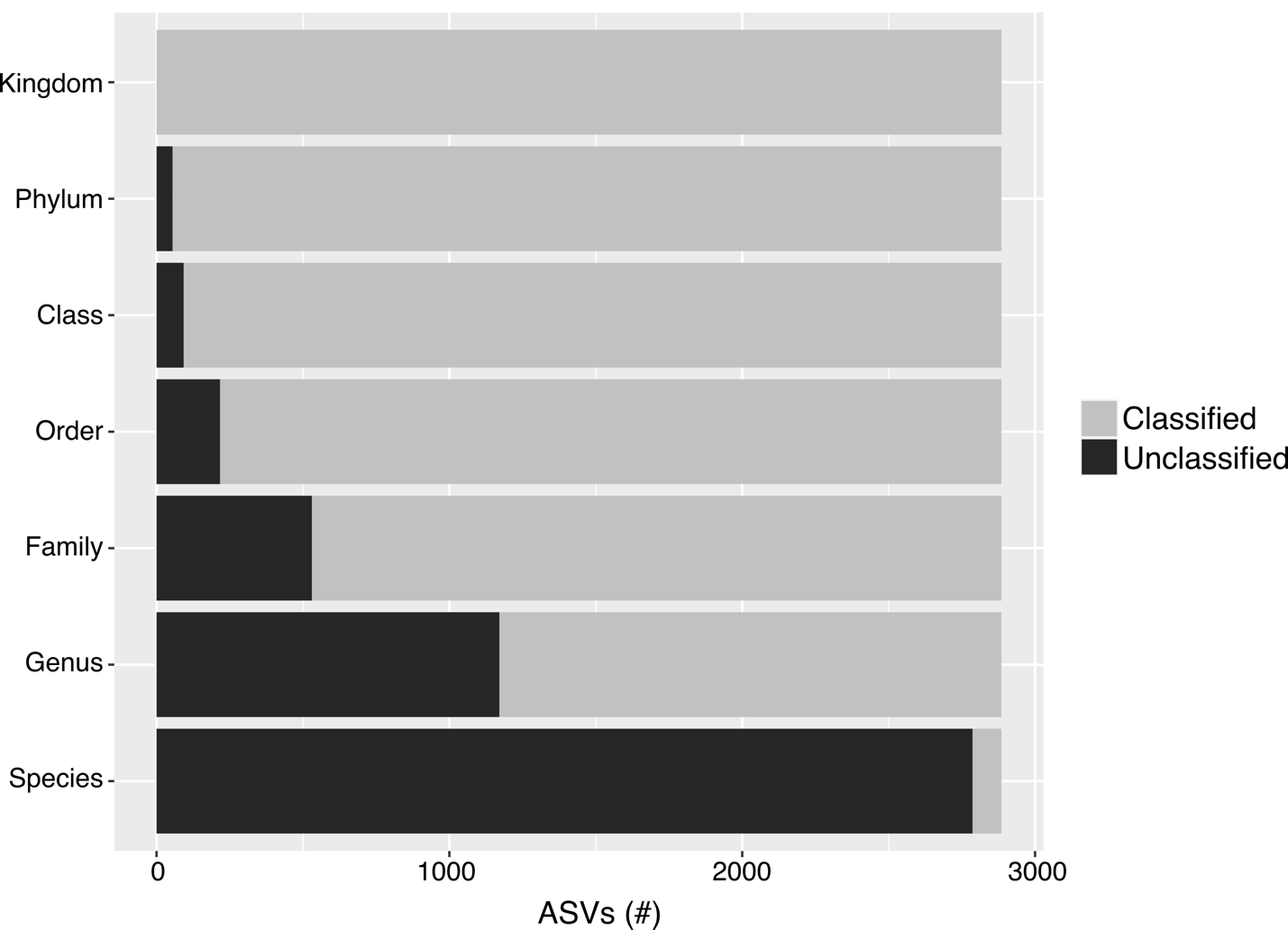


**Figure S7.** Level of taxonomic novelty of ASVs (2,885 ASVs in total). The number of classified and unclassified ASVs at each taxonomic level was determined according to rank assignment and taxonomic classification in DADA2 using the AssignTaxonomy function and the SILVA database.


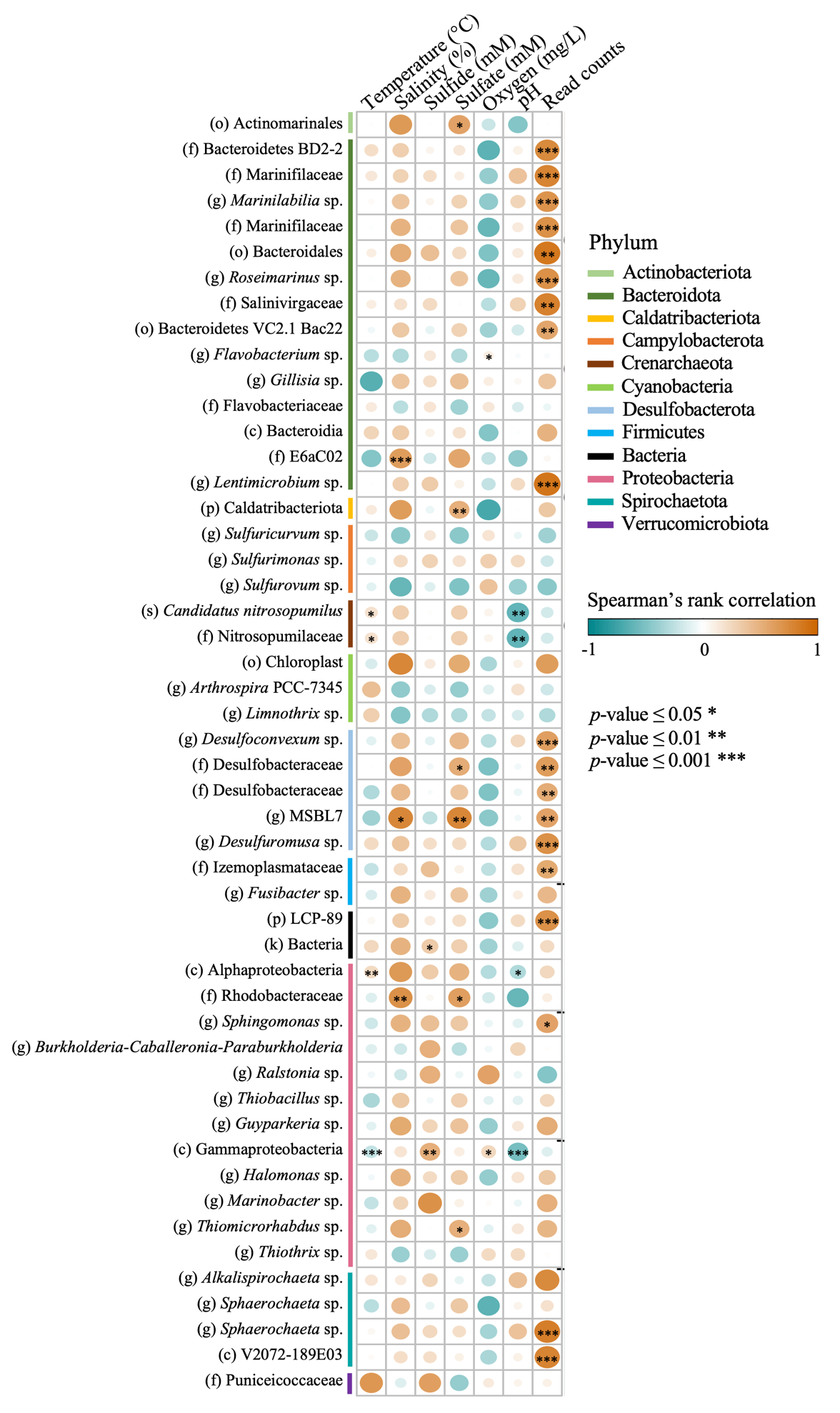


**Figure S8.** Spearman’s rank correlation of the top 50 most abundant taxa in the subset of thirteen 16S rRNA gene sequencing data sets with environmental parameters. Metadata for this plot is located in Table S4.


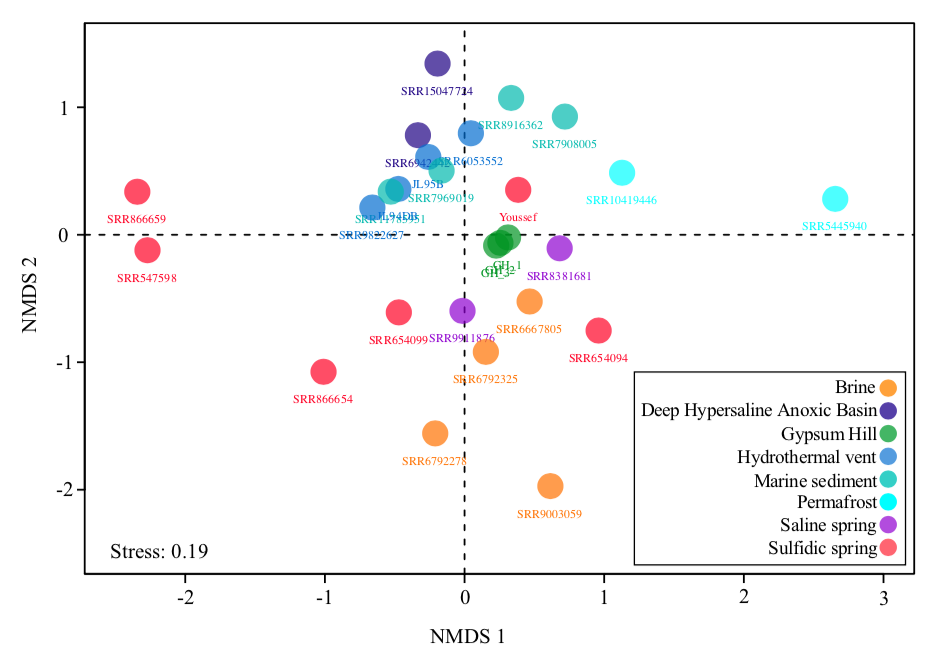


**Figure S9.** NMDS plot with Bray-Curtis dissimilarity matrix for 16S rRNA gene amplicon sequences from GH and comparable environments. Metadata for this plot is located in Table S4.

**Table S1 (xlsx).** Physical and chemical parameters in GH-4. Located in Supplementary Tables xlsx file.

**Table S2.** Sequencing library statistics.

| **Library type** | **Sample Name** | **Sample NCBI ID** | **Read length (base pairs)** | **Raw read pairs** | **Read pairs after quality control** | **Read mapping rate to metagenome co-assembly (%)** |
| --- | --- | --- | --- | --- | --- | --- |
| Metagenome | Replicate 1 (GH1) | SAMN32378173 | 2x100 | 34485105 | 34483207 | 71.52 |
| Metagenome | Replicate 2 (GH2) | SAMN32386946 | 2x100 | 37844336 | 37843584 | 80.54 |
| Metagenome | Replicate 3 (GH3) | SAMN32386947 | 2x100 | 36789239 | 36788071 | 81.09 |
| Metatranscriptome | Replicate 1 (R2) | SAMN32386639 | 2x100 | 59259431 | 30221082 | 59.09 |
| Metatranscriptome | Replicate 2 (R5) | SAMN32386637 | 2x100 | 29110743 | 18697235 | 30.72 |
| Metatranscriptome | Replicate 3 (R6- technical replicate) | SAMN32386638 | 2x100 | 48840940 | 25313326 | 60.72 |
| Metatranscriptome | Replicate 3 (R9- technical replicate) | SAMN32386638 | 2x100 | 48405899 | 29204507 | 42.83 |
| Amplicon | Replicate 1 (GH34) | SAMN32406239 | 2x300 | 322476 | 170529 | N/A |
| Amplicon | Replicate 2 (GH35) | SAMN32406240 | 2x300 | 256138 | 130640 | N/A |
| Amplicon | Replicate 3 (GH36) | SAMN32406241 | 2x300 | 474775 | 202598 | N/A |

**Table S3.** Metagenome co-assembly statistics.

| Contigs | 464165 |
| --- | --- |
| Total length (bp) | 519632022 |
| Maximum contig length (bp) | 874936 |
| Average contig length (bp) | 1119 |
| N50 (bp) | 2577 |
| Contigs >2 kb | 43091 |
| Total length contigs >2 kb | 283840215 |
| Metagenome-assembled genomes (#) (>50% complete, <10% contamination) | 57 |
| Total length in metagenome-assembled genomes (bp) | 182741013 |

**Table S4 (xlsx).** Metadata for amplicon metagenome libraries used in beta diversity analysis. Located in Supplementary Tables xlsx file.

**Table S5 (xlsx).** Taxonomic count table used for beta diversity analysis. Located in Supplementary Tables xlsx file.

**Table S6 (xlsx).** List of contigs in each MAG. Located in Supplementary Tables xlsx file.

**Table S7 (xlsx).** MAG supplemental information. Located in Supplementary Tables xlsx file.

**Table S8 (xlsx).** Taxonomic classification of genes of interest with mapped transcripts. Located in Supplementary Tables xlsx file.

**Table S9 (xlsx).** Gene content of MAGs. Located in Supplementary Tables xlsx file.

**Table S10 (xlsx).** Relative expression of genes of interest in MAGs. Located in Supplementary Tables xlsx file.

**Table S11 (xlsx).** Total tpm per genome feature product ID. Located in Supplementary Tables xlsx file.

**Table S12 (xlsx).** Gene counts and relative expression of genes of interest in the metagenome. Located in Supplementary Tables xlsx file.

**Table S13 (xlsx).** Complete BLAST output of elemental sulfur reduction proteins queried against *Desulfuromusa* sp. GH17. Located in Supplementary Tables xlsx file.

**Table S14 (xlsx).** Relative expression of all genes. Located in Supplementary Tables xlsx file.
